# Optical coherence photoacoustic microscopy for 3D cancer model imaging with AI-assisted organoid analysis

**DOI:** 10.1038/s41377-025-02177-2

**Published:** 2026-02-05

**Authors:** Abigail J. Deloria, Agnes Csiszar, Shiyu Deng, Mohammad Ali Sabbaghi, Francesco Branciforti, Lukasz Bugyi, Giulia Rotunno, Richard Haindl, Rainer Leitgeb, Massimo Salvi, Manojit Pramanik, Yi Yuan, Leopold Schmetterer, Gergely Szakacs, Wolfgang Drexler, Kristen M. Meiburger, Mengyang Liu

**Affiliations:** 1 Center for Medical Physics and Biomedical Engineering, Medical University of Vienna, Vienna, Austria; 2 Center for Cancer Research, Medical University of Vienna, Vienna, Austria; 3 PolitoBIOMed Lab, Biolab, Department of Electronics and Telecommunications, Politecnico di Torino, Torino, Italy; 4 Department of Electrical and Computer Engineering, Iowa State University, Ames, IA USA; 5 School of Electrical Engineering, Yanshan University, Qinhuangdao, Hebei, China; 6 Department of Clinical Pharmacology, Medical University of Vienna, Vienna, Austria; 7 Singapore Eye Research Institute, Singapore, Singapore; 8 SERI-NTU Advanced Ocular Engineering (STANCE) Laboratory, Singapore, Singapore; 9 Ophthalmology and Visual Sciences Academic Clinical Program, Duke-NUS Medical School, Singapore, Singapore; 10 Institute of Molecular and Clinical Ophthalmology Basel, Basel, Switzerland; 11 School of Chemistry, Chemical Engineering and Biotechnology, Nanyang Technological University, Singapore, Singapore; 12 Fondation Ophthalmologique Adolphe De Rothschild, Paris, France

**Keywords:** Biophotonics, Photoacoustics, Microscopy

## Abstract

Cancer organoids and cancer spheroids are 3D cell culture models with distinct yet overlapping purposes in cancer research. Various commercially available optical imaging techniques have been employed to study these cell cultures, but these methods suffer from various limitations such as the requirement of fluorescence labeling, complicated sample handling, and limited image volume size. In this work, we demonstrate a multimodal optical coherence photoacoustic microscopy (OC-PAM) system for the study of these models, overcoming these limitations. We first performed a longitudinal study using optical coherence microscopy (OCM) for breast cancer organoids. Using the OCM imaging results, artificial intelligence (AI)-based algorithms were developed to automatically segment individual organoids and classify their viability over time using a radiomics texture feature approach, enabling robust, quantitative tracking and classification at the single-organoid level. To supplement OCM’s contrast, we then performed OC-PAM imaging of spheroid models with both melanin positive and melanin negative cells. In the second study, the OC-PAM images clearly mapped the distribution of melanin positive cells hidden amongst melanin negative cells. These results suggest that OC-PAM coupled with AI techniques can be a powerful tool to study cancer organoids and cancer spheroids.

## Abstract (web summary)

Optical coherence microscopy with AI assisted algorithms is used to track organoids and their viability longitudinally. Photoacoustic imaging adds rare cell detection function to the 3D cell culture analysis toolbox.

## Graphical abstract

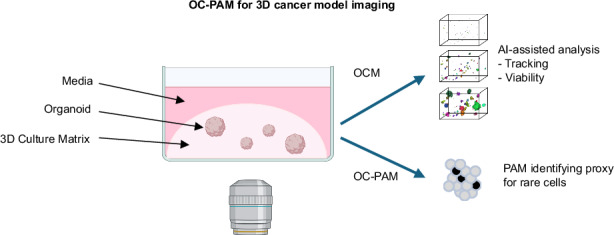

## Introduction

Cancer organoids and cancer spheroids are 3D cell culture models established from patient or animal tumors. They bear similarities to the original tumor tissue and can resemble the complexity of tumors both structurally and functionally^1^. As these models can be generated in a time- and cost-effective manner, they have become an indispensable preclinical cancer model and are widely used in a variety of studies from carcinogenesis, drug development, to personalized medicine^2,3^. Although sometimes used interchangeably and manifesting overlapping features, cancer organoids and cancer spheroids have distinctive purposes and require different cellular sources as well as establishment protocols^1^. Since cancer organoids are histologically and genetically close to the original tumor tissue, they are exploited in a wide array of preclinical cancer research. In comparison, cancer spheroids are relatively less complex and can be assembled directly from cancer cell lines. As of now, cancer organoids as well as cancer spheroids have been established for a myriad of cancers such as, but not limited to, malignancies of the brain^4,5^, breast^6,7^, lung^8,9^, colon^10,11^, and pancreas^12,13^.

Cancer organoids and spheroids underwent an exponential increase of impact partially attributable to their nature of being easily observable by common imaging tools. For these in vitro models established in transparent wells, brightfield and fluorescence microscopy can be readily used for observation^14–17^. A few examples for brightfield microscopy in organoid imaging include its use in distinguishing morphologies of organoids^14^, prediction of cell membrane and nucleus, and the tracking of the viability of organoids^18^. Immunofluorescence and immunohistological 2D imaging of tissue sections are also standard methods to study the cellular composition and spatial distribution of cells in the organoids and the spheroids^19,20^. As researchers probe deeper into the underlying mechanisms of cancer which are explorable through organoids and spheroids, more advanced imaging techniques such as confocal microscopy^21^, multiphoton microscopy^22^, and light-sheet microscopy^23^ have been recruited with the ability of 3D imaging and quantitative analysis of biomarkers on a cell-by-cell basis^24^. These above-mentioned imaging methods, however, all come with their own limitations. For example, brightfield microscopy has low resolution and is not suitable for 3D imaging. This means that organoids that are stacked above each other cannot be properly distinguished, therefore limiting brightfield microscopy’s application in high-throughput imaging. Confocal fluorescence microscopy and multiphoton microscopy may experience issues in phototoxicity and are slow in image acquisition. For light-sheet microscopy, the imaging system requires special sample holders and is therefore challenging to use when high-throughput imaging is needed. Furthermore, the cellular composition visualized by these fluorescence-based methods requires staining differentiation markers and proteins of interest^16,17,25^, making these microscopy methods an end-point analysis tool and inherently incompatible with longitudinal tracking. Additionally, the use of fluorescence labeling may experience photobleaching, hence affecting the accuracy and reliability of the acquired data^19^. In view of these limitations, an ideal imaging solution for 3D cancer cultures should be able to provide label-free and volumetric images with sub-cellular resolution in a non-destructive manner. The solution should also be able to perform longitudinal imaging with easy sample handling and provide both structural and functional information for the 3D cultures at fast speed.

As a non-invasive and non-contact optical imaging modality, optical coherence microscopy (OCM) features high speed, millimeter level imaging depth, and subcellular resolution in a label-free setting. Its contrast arises from the inherently different optical scattering properties of biological tissues^26,27^. OCM has been proved useful in the characterization of several organoid models, demonstrating good correlation with fluorescence microscopy (FLM)^28^. Longitudinal monitoring of cancer spheroid growth by OCM enabled quantification of general volume changes, differentiating between live cells and the necrotic core^29^. Implementing the OCM system in an inverted microscope geometry, high-throughput imaging of patient-derived cancer organoids was achieved. Using this system, drug treatment response has been assessed based on single-organoid tracking combined with volume measurement^30^. The deep penetration and large volume imaging capabilities of OCM have been exemplified when it was applied to image human placenta-derived trophoblast organoids in situ, revealing the multiple cavities in the organoids characteristic of the differentiation status^31^. Finally, the high-speed feature of OCM has been demonstrated through real-time imaging of human heart organoids, providing information on beating patterns and chamber structure development^32,33^. Furthermore, based on logarithmic intensity variance, dynamic optical coherence tomography (D-OCT) could visualize intracellular motion. This new label-free motion contrast has been then demonstrated in human derived cancer spheroid imaging, successfully assessing drug-response patterns to anti-cancer drugs^34,35^.

Despite all the advantages of OCM, its contrast mechanism still lacks functional and molecular sensitivity^19^, and therefore limits its application to mostly morphogenesis studies. Integrating other imaging modalities with complementary features enables the extraction of more comprehensive information. Among non-invasive optical imaging techniques, photoacoustic microscopy (PAM) has been shown to offer optimal complementarity to OCM^36^. PAM uses optical absorption as the contrast mechanism and has been applied in a plethora of preclinical and clinical settings^37–39^. A specific implementation of PAM, termed optical resolution PAM (OR-PAM), has been shown to have similar resolution, speed, and volume as those of OCM^40–42^. A combined optical coherence PAM (OC-PAM) system is hence of great interest also in cancer organoid and cancer spheroid imaging. As for PAM alone, despite many successful demonstrations in translational research, its use in 3D cellular imaging remains scarce^43^. One example is to use PAM in pancreatic-stromal cancer spheroid imaging, showing its ability to examine nanoparticles’ behavior inside the spheroids^44^.

Another crucial factor when considering organoid imaging is the need to obtain quantitative data from the imaging result. Given that many organoids are often grown within one well, manual analysis to evaluate growth patterns is tedious and impractical, underlining the need for automatic segmentation and quantitative analysis methods to extract important biological information. Previous studies have shown the feasibility of automatically segmenting and quantifying organoid growth in OCM images through a structural and morphological analysis^45^. Going beyond morphological analysis, radiomics texture features have shown to differentiate tissue types and have been amply used in clinical imaging modalities for cancer identification^46,47^, but are yet to be fully explored with organoid viability analysis with OCM. Recently, automatic segmentation of organoids and quantitative assessment of critical parameters, such as size, area, volume, and cardiac beating has been reported^48^, however, without including in-depth texture feature analysis. Furthermore, the potential of artificial intelligence (AI) tools is becoming more and more evident in cancer diagnosis and theranostics applications^49,50^, and in general for advancing healthcare^51^. Recent works have also focused on how AI can be employed for label-free microscopy and for deep tissue neurophotonics^52,53^.

In this work, an OC-PAM system was devised and equipped with an AI-assisted image analysis package. The functionality and features of the system are demonstrated stepwise in carefully designed experiments. First, to show the system’s capability of longitudinal imaging and its value in drug screening, the OCM mode was used to batch image breast cancer organoids over a period of up to 21 days to evaluate the organoids’ drug response to chemotherapy. Secondly, to go beyond organoid tracking and volumetric analysis, we demonstrate a radiomics-based approach to automatically classify organoids’ viability in a label-free manner. This innovative translation of radiomics into organoid characterization adds a functional layer to the information extractable from OCM. Thirdly, knowing that molecular contrast is also critical in 3D cancer model imaging, PAM is introduced as a complementary imaging modality to OCM. Inspired by the PAM imaging study of neuromelanin containing human midbrain organoids^43^, murine melanoma and breast cancer cells were co-cultured to model 3D multicellular structures containing rare cells with distinctive features detectable by OC-PAM. In the future, the AI-assisted OC-PAM imaging solution will enable the monitoring of drug responses across the entire organoid populations down to single cells, guiding the development of more effective combination therapies targeting both bulk tumors and rare surviving cells.

## Results

**Fig. 1:**
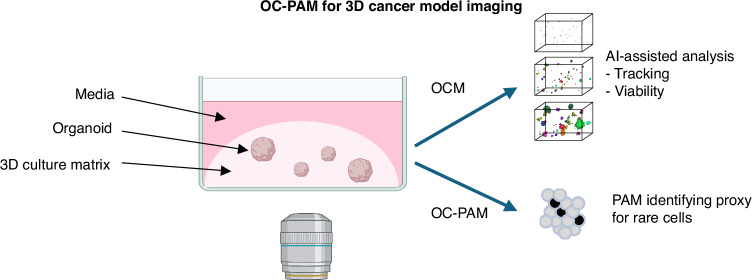
Conceptual schematic for the study

Three experiments were designed to illustrate OC-PAM’s multiple features, which are fit to deal with different research challenges using 3D cancer models. The conceptual schematic for the study is shown in Fig. 1.

### Longitudinal imaging of breast cancer organoids

**Fig. 2:**
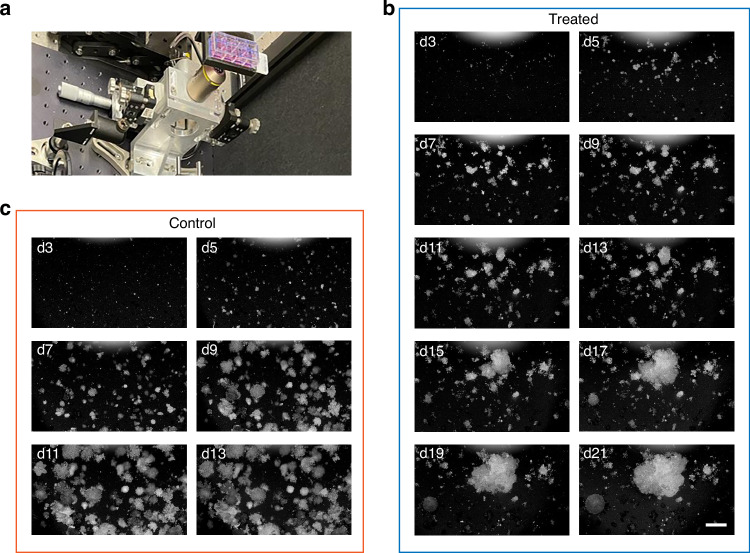
Longitudinal OCM imaging of breast cancer organoids. **a** Photo showing an 8-well plate placed on top of the OC-PAM scanner. **b** *En face* images of the carboplatin-treated group from day 3 to day 21. **c** *En face* images of the control group from day 3 to day 13. All images are presented in logarithmic scale using the standard deviation intensity projection. Scale bar: 400 µm

To demonstrate the functionality of OCM, carboplatin-treated breast cancer organoids of the KB1P model^54^ were monitored longitudinally. Prior to the longitudinal study, various cell seeding densities were tested, as reported in the supplementary material (Fig. S1). Ultimately, one thousand cells were seeded in a 10 µL matrix, overlaid with culture media and monitored every other day. Figure 2a shows the 8-well organoid culture plate used in the experiment. Representative *en face* images of the drug-treated groups and the untreated control are shown in Fig. 2b and c, respectively. While control organoids grew rapidly, most organoids in the treated group stayed small, except for a few that significantly increased in volume and merged by day 21. The control group imaging stopped on day 13, because we noticed that on day 15 most of the organoids in the control group had merged.

**Fig. 3:**
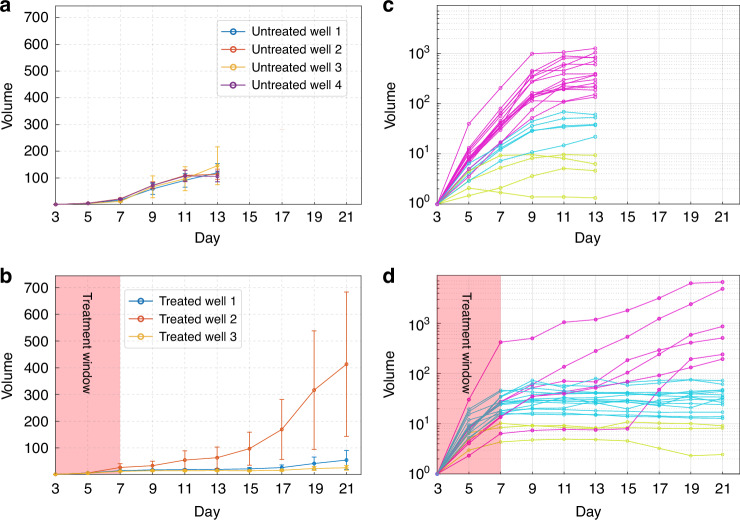
Average volume growth analysis of OCM imaging of breast cancer organoids. **a** Average volume growth of all untreated organoids in four selected wells. **b** Average volume growth of all treated organoids in three selected wells. **c** Volume growth of individual untreated organoids shown on a logarithmic scale, utilizing the same color scheme for growth patterns: (pink lines) rapid growth, (light blue lines) medium growth, and (yellow lines) weak or no growth. **d** Volume growth of individual treated organoids displayed on a logarithmic scale, demonstrating three distinct growth patterns: (pink lines) rapid growth, (light blue lines) medium growth, and (yellow lines) weak or no growth. All volume measurements were normalized to the initial volume on day 1, resulting in dimensionless values. The y-axis for (**c**, **d**) are portrayed in a logarithmic scale due to the large differences in volume changes for the single organoids

Quantitative analysis of average volumes clearly shows the reduced growth rate of drug-treated organoids as demonstrated in Fig. 3. The relatively large variance in the growth of the individual organoids (shown in Fig. 3c and d) is mainly due to two factors: (i) some of the segmented organoids in both groups show little change in volume; (ii) some of the segmented organoids in the treated group show a distinct pattern of slow growth in the initial few days after drug withdrawal followed by a rapid expansion and fusion with other organoids, resulting in a very large increase in volume. Transient quiescence followed by renewed proliferation is characteristic of drug tolerant persister (DTP) cells^55^. The proportions of treated organoids exhibiting the typical DTP cell growth pattern were 4.17%, 10.0%, and 6.25% in treated wells 1, 2, and 3, respectively, whereas the majority demonstrated the expected response to treatment, indicated by a sustained arrest in growth. Figure 3a and b show the average volume growth of all tracked organoids in the different wells for the untreated and treated organoids, respectively. Figure 3c and d display a subset of the individual growth patterns of organoids within the different wells. The untreated wells generally show continuous growth, though with varying dynamics. Some exhibit rapid expansion, with an approximately ten-fold increase in relative volume between day 3 and day 5, followed by a further rise of two to nearly three orders of magnitude by day 9 (pink lines in Fig. 3c). Others display more contained growth, reaching almost two orders of magnitude by day 13 (light blue lines). A subset shows only limited growth, with relative volume increasing by about one order of magnitude by day 13 (yellow lines). In contrast, a number of organoids in the treated volume present a slow and stable growth, demonstrating on average a ten-fold relative volume increase between day 3 and day 7. This growth then plateaus after day 7 or 9 (yellow and light blue curves in Fig. 3d), remaining essentially flat on a logarithmic scale, reflecting that any relative volume increases between time points are negligible compared to order-of-magnitude changes. However, the organoids which exhibit the typical DTP cell growth pattern (pink lines in Fig. 3d) typically portray a halting growth pattern between day 7 and day 13 and then continue to grow in a rapid manner, with relative volume increases exceeding three orders of magnitude.

**Fig. 4:**
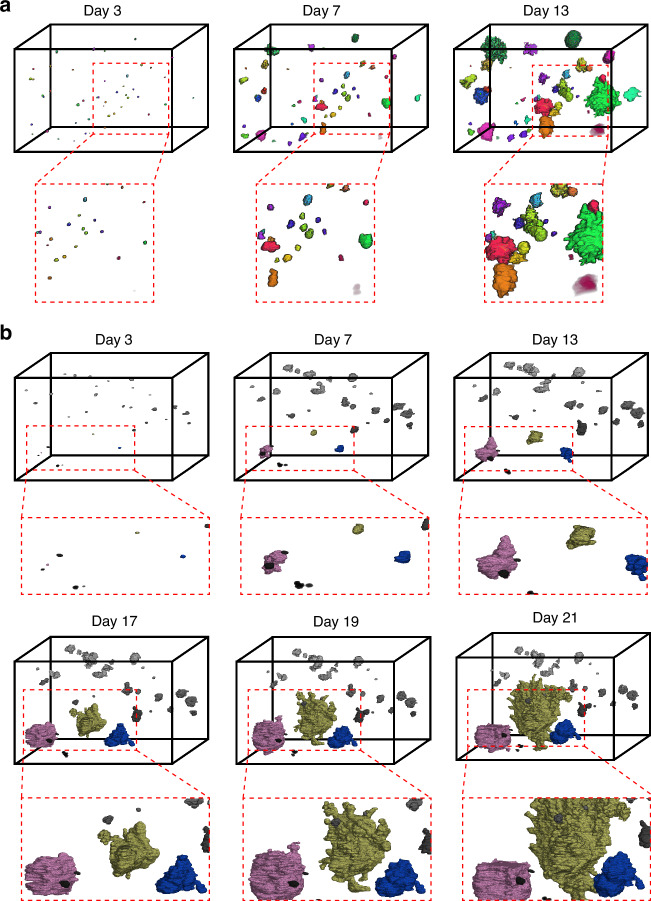
Example volumes of the untreated and treated organoids over multiple time points. **a** Example volume of untreated organoids over three time points showing overall rapid and consistent growth patterns. **b** Example volume of treated organoids over six time points. Three organoids (colored in pink, yellow, and blue) show a DTP-like growth pattern. The images were obtained through the volume rendering of the automatic segmentations using the open-source 3D Slicer tool

Figure 4 provides a visual representation of the distinct growth patterns observed in untreated and carboplatin-treated breast cancer organoids over the same first three time points (i.e., day 3, day 7, and day 13). The treated wells are shown over 3 more time points: day 17, day 19, and day 21, to illustrate the rapid growth of a few organoids, whereas most of the organoids remain small and do not continue to consistently grow. Figure 4a showcases organoids from the untreated control group, displaying consistent and rapid growth across all time points. In Fig. 4b, three organoids from the treated group are highlighted with three different colors (i.e., blue, yellow and pink), with the yellow one especially exhibiting the characteristic growth pattern of DTP cells. The gray organoids present in the well, on the other hand, appear to grow in a limited fashion until day 13 and then do not portray any noticeable growth in volume in the subsequent days. These examples provide a clear visual comparison of the growth patterns described quantitatively in Fig. 3, emphasizing the differences between treated and untreated organoids and the heterogeneity of treatment response within the treated group. Additional capabilities of the algorithm to characterize organoid growth phenomena, such as the merging of organoids, are reported in the supplementary material (Fig. S2).

### Classification of organoid viability

**Fig. 5:**
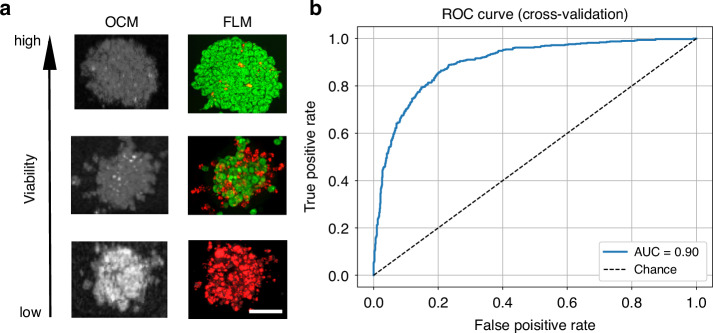
Differentiation between live and dead organoids via radiomics-based classification of OCM data. **a** Example OCM images of unlabeled organoids and subsequent FLM images after acridine orange (green channel for living cells) and propidium iodide staining (red channel for dead cells) of the same organoids for viability classification. Scale bar: 50 µm. **b** Receiver operating characteristic (ROC) curve for the XGBoost classifier obtained on 10 folds

Classification of organoids based on viability and response to treatment is crucial for assessing drug efficacy and understanding cancer progression. Following OCM scanning, organoids were therefore labeled by acridine orange and propidium iodide to independently distinguish live and dead cells by fluorescence microscopy (Fig. 5a), which were employed as the ground truth for viability status classification after cross-referencing the same organoids on both the FLM and the OCM images. This required a maintained position of organoids within the 3D matrix, thereby precluding the use of destructive staining methods, such as histopathological staining, that are otherwise commonly applied for organoid characterization (Fig. S3).

The classification performance based on OCM data was evaluated using a receiver operating characteristic (ROC) curve analysis (Fig. 5b). The area under the ROC curve (AUC) was then computed as the average across the 10 cross validation folds of the XGBoost classifier. The obtained AUC was equal to 90%, indicating that there is a 90% chance that the classifier will assign a higher score to a randomly chosen positive (i.e., high viability) organoid than to a randomly chosen negative (i.e., low viability) organoid. To evaluate the classifier performance changes with different training set sizes, we employed the same ROC curve analysis using only a subset of the entire dataset, ranging from 10% to 100%, with a step size of 10%. The results are shown in Fig. S4. The nine most important texture features were found to be: ‘Sum Entropy’, ‘Run Percentage’, ‘Joint Entropy’, ‘Difference Entropy’, ‘Run Length Non-Uniformity Normalized’, ‘Autocorrelation’, ‘Joint Average’, ‘Gray Level Variance’, and ‘Run Entropy’. On one hand, the results show the importance of first-order texture features based on the intensity histogram properties. On the other hand, they also underline the importance of not only considering the overall distribution of intensity values but also the spatial localization and patterns between pixels, i.e., the second-order texture features of the OCM images. These classification results demonstrate that the radiomics approach based on OCM imaging provides a means for non-invasive and non-destructive analysis of organoid viability over time.

The trained classifier was then implemented and tested on the longitudinal organoid data. Texture features were then extracted from each of the tracked organoids and the trained XGBoost classifier was employed to classify the organoids as either high or low viable at the different time points based solely on the OCM data. The ground truth fluorescence data is not available for these samples, as fluorescence labeling may have altered growth and viability of organoids. Hence, complete validation of the classification results on the tracked organoids is not possible. Still, the preliminary results provided by the classifier are encouraging. Of the tracked organoids in the well portrayed in Fig. 4b, 37% presented an overall viability status as lower than 50% (i.e., the classifier predicted a low viability for at least half of the considered time points). Further, organoids which halted their growth were categorized by the classifier being of overall low viability. In contrast, the organoids that are displayed in pink and yellow in Fig. 4b were classified as having an overall viability equal to 80% and 70%, meaning that the classifier predicted the organoid as having a high viability at 8 and 7 time points, respectively, out of the 10 considered time points. The other organoid that presents a DTP-like growth pattern in Fig. 4b presented a lower overall viability status, equal to 40%, which could be because this organoid shows a more plateaued growth in the middle days, when their texture was more similar to low viability organoids than to high viability organoids. The well portrayed in Fig. 4a, on the other hand, shows an overall viability status of all organoids equal to 80%. A visualization and discussion of the overall organoid well viability evolution over time and of specific organoids are included in the supplementary material (Fig. S5).

### Complementing OCM with PAM for rare cell detection in 2D cell cultures

**Fig. 6:**
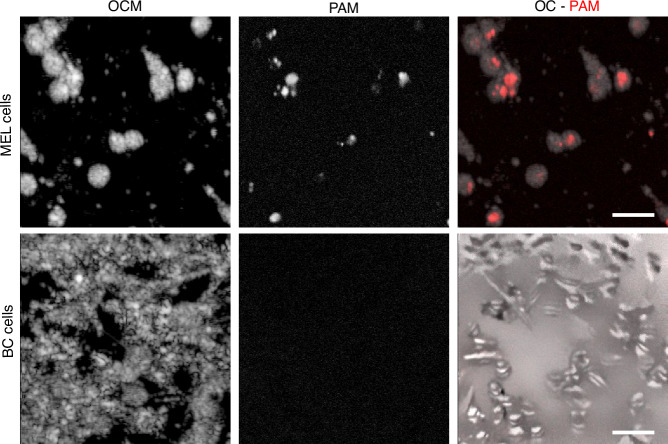
OC-PAM imaging of 2D cell cultures of melanoma and breast cancer cells. *En face* view of melanoma cells (upper row) and breast cancer cells (lower row) for OCM, PAM, and OC-PAM. MEL melanoma, BC breast cancer. Scale bar: 50 µm

To evaluate the feasibility of OC-PAM in the detection of rare cells, various ratios of melanoma cells (B16-F10) were mixed with breast cancer cells without melanin expression (4T1). Figure 6 shows *en face* views of superimposed OCM and PAM data of 2D cell cultures of melanoma cells (upper row) and breast cancer cells (lower row). OCM images show morphological information of the 2D cell cultures, whereas PAM images reveal the melanin content. PAM depth profiles corresponding to single cells indicate that 4T1 cells generate no PAM signals whereas melanoma cells yielded PAM signals. To assess the relationship between PAM signal intensity and melanin concentration, we imaged tubes filled with melanin solutions of different concentrations, whose results are presented in Fig. S6.

### OC-PAM spheroid imaging

**Fig. 7:**
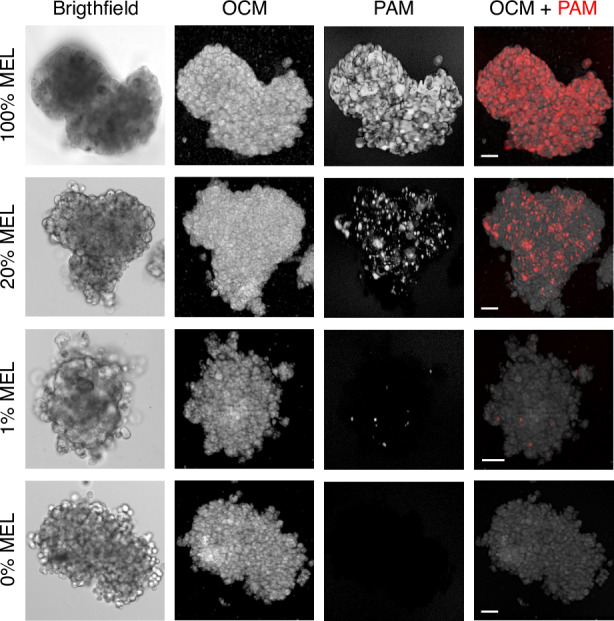
Representative images of cancer spheroid models. Melanin-positive B16-F10 melanoma (MEL) cells were mixed with 4T1 breast cancer cells in the indicated proportions to form spheroids. The leftmost column shows the 2D images acquired using brightfield microscopy. OCM *en face* images are shown in logarithmic scale using standard deviation intensity projections. PAM *en face* images are shown in maximum intensity projections in logarithmic scale. Scale bars: 50 µm

**Fig. 8:**
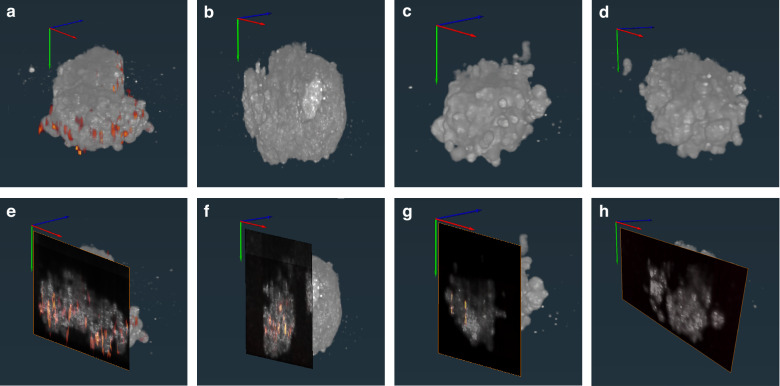
3D rendering of cancer spheroid models with different percentages of melanin positive cells. Spheroid model with **a** 100%, **b** 20%, **c** 1%, **d** 0% melanoma (melanin positive) cells and with corresponding cross-sections (**e**–**h**) to demonstrate the melanin distribution inside the spheroids (PAM: orange-red, OCM: gray). Videos of these 3D renderings are available in the supplementary material

Next, the two cell lines were used to form spheroid co-cultures with increasing melanin-positive cell content. Figure 7 shows the *en face* views from brightfield microscopy, OCM, PAM, and PAM superimposed on OCM, respectively. Brightfield microscopy and OCM both convey morphological information of the imaged 3D cell cultures, but a direct comparison among these three modes demonstrates that OCM outperforms brightfield microscopy in revealing the fine structures down to individual cell level, which is even better illustrated in the 3D perspective views shown in Fig. 8. From the rightmost columns of Fig. 7 and from Fig. 8, melanoma cells can be distinguished with high clarity and contrast from the morphological background. Spheroids exclusively consisting of melanoma cells yield overlapping PAM and OCM images. The PAM signal amplitude is proportional to the concentration of melanin. In spheroids composed of 20% melanoma cells, clusters of melanoma cells and individual melanoma cells can be visualized (note that the depth profiles are projected onto a single plane). Sparsely distributed melanin-positive cells are successfully identified even at 1%, indicating that OC-PAM can accurately locate rare cells in 3D structures. Spheroids lacking melanoma cells show no PAM signal, confirming that melanin is the only contrast available for PAM in the experiment.

## Discussion

An OC-PAM system, equipped with AI-assisted analysis algorithms, has been developed and demonstrated in the imaging of cancer organoids and cancer spheroids. Being non-invasive, non-contact, and label free, OCM can be used in longitudinal studies of cancer organoids under chemotherapeutic treatment with no perturbation to growth kinetics. The high imaging speed makes the acquisition of a single volume in just 84 s, allowing high-throughput batch imaging. Though organoid growth analysis using OCM has been shown before^30^, the algorithm developed in this work does more–it tracks each organoid in a pool of organoids automatically. Furthermore, this study demonstrates for the first time the feasibility of a deep learning-based automatic segmentation and single organoid tracking in OCM volumes over an extended 21 days, quantitatively showing the growth retardation effect of the treatment on individual 3D structures. Differential quantitative volumetric tracking is extended with a radiomics-based analysis on the viability of each structure, providing the likelihood of “live” or “dead”. These features together offer fast and inert monitoring of 3D cultures exposed to any selection pressure and enable a comprehensive analysis of volumetric changes over time with a categorized measure on viability throughout a dynamic scale from population level down to individual organoids. The combined OC-PAM goes beyond, and empowers sub-structural analysis at cellular resolution by enabling the detection and quantification of rare cells surviving a selection pressure, assuming they bear detectable contrast for PAM. This feature provides the foundation for ample future applications, like monitoring the survival of rare cells after exposure to near-death challenges, e.g., cancer therapy, or performing cooperativity studies between dying and surviving cancer cells in a selective environment. While within the frame of this work the OC-PAM proof-of-concept study on detecting rare cells in 3D structures and the high-performance AI-assisted OCM approach for longitudinal drug-response monitoring remained two separate sets of experiments, future work envisions the combination of these two powerful tools to enable quantitative monitoring of drug response from bulk population to single cells, including the detection of rare cells that survive drug treatment in dying organoid structures. This ensuing study will provide robust throughput with high quantitative accuracy for sub-population studies simultaneously delivering valuable insights into the mechanisms of drug resistance and guiding the development of more effective combination therapies that target both the bulk tumor population and rare surviving cells.

In our OCM analysis, the relative occurrence of distinctive growth kinetics of individual organoids can provide a deep characterization about the efficacy of drug responses and the clonal evolution of drug resistance with high clinical relevance. Using individual tracking, three main growth kinetic patterns were delineated: (i) a continuous increase in volume reflecting exponential growth; (ii) structures without volumetric changes, representing no growth; (iii) organoids with a biphasic “stalled-then-renewed” growth resembling DTP-like behavior. While the first two categories were found in both untreated and treated cultures, the biphasic growth kinetics was an exclusive drug-induced phenomenon. This indicates that while the vast majority of cancer cells were responsive to therapy, rare clones with resistance to the applied doses or in an earlier phase of drug tolerance could be identified, which had been staying hidden in bulk analytic methods. This quantitative depth will have a high impact on drug development and drug efficacy screening.

Moreover, here we demonstrate for the first time how an AI radiomics-based texture feature approach to organoid analysis in OCM volumes can effectively differentiate organoid viability. The growth kinetics of organoids matched well with the classifier. This result is promising as it demonstrates that information on the health status of an organoid can be effectively extracted from the texture features of the organoids based solely on non-destructive OCM data. Further studies are warranted to confirm these findings, and to add more viability ranges. Indeed, a more comprehensive and large dataset is essential for further validation and generalization of the approach, which is planned for future studies. We will also focus on integrating additional modalities and labels to increase classifier sensitivity and to reduce ambiguity for the reference data used to validate the classifier both in training and validation stages. Nonetheless, the radiomics-based approach for organoid viability differentiation demonstrates the potential for non-invasive, high-throughput screening of drug efficacy and toxicity in 3D cancer models, which could accelerate the drug discovery and development process.

Despite the potential impact of OCM in drug screening using cancer organoid models, in this work, its contrast is only from optical scattering, limiting the visualization to morphology. Recently, the introduction of D-OCT to 3D cell culture imaging has shed light on revealing functional information from OCM at sub-cellular resolution^34,35^. Applying the logarithmic intensity variance and the late optical coherence tomography correlation decay speed algorithms, cancer spheroid dynamics were successfully extracted, indicating different action mechanisms of the anti-cancer drugs. Since these algorithms do not require modifications of the optical design of the OCM system, adding a D-OCT function to the OC-PAM system may bring additional valuable dynamic contrast for cancer organoid and cancer spheroid imaging.

Whereas the OCM mode, along with the automatic tracking and viability identification algorithms, can track morphological changes in 3D cell cultures, differentiating cell types is challenging for OCM due to the lack of contrast mechanisms. The results shown in Figs. 6, 7, 8 clearly indicate that adding PAM to OCM can bring valuable absorption-based contrast to the co-registered image. In the cancer spheroid OC-PAM imaging experiment, we used melanin as the endogenous contrast for PAM. Single-cell level detection sensitivity of PAM was shown. The fused OC-PAM images, especially the ones for the 1% melanoma spheroids, demonstrate the capability of OC-PAM to locate rare cells inside 3D cellular structures. This feature of OC-PAM makes it a powerful tool to study DTPs or other rare cell populations, which can in turn exert a big impact on the research of drug-resistant mechanisms in cancer with earliest capture of rare clonal expansions^56^. As a pilot study, melanoma cells were mixed into breast cancer spheroids at low ratios as a surrogate of rare cells, using melanin as the proof-of-principle contrast for PAM. Nevertheless, melanin expression can be forced by engineered overexpression of tyrosinase, the critical converting enzyme of tyrosine to melanin, in diverse cancer cells^57^. In addition, more applicable in translational research, there are bountiful contrast agents available for PAM in molecular imaging^58^. We believe that the use of biofunctionalized contrast agents can make OC-PAM capable of differentiating many different types of cancer cells in 3D cell cultures. In this work, we only used a single excitation wavelength for PAM. When multiple absorbers or chromophores are present, spectroscopic PAM can be easily implemented to provide quantitative analysis of the absorber concentrations^59^.

The current configuration of the OC-PAM system still relies on a transducer to acquire the photoacoustic signal. This implementation limits PAM to working in transmission mode only. The insertion of the transducer into the culture may also unintentionally bring contamination. Since we currently use commercial 8-chamber slides as sample holders, reflection mode PAM is difficult to implement. The use of flexible fiber optic photoacoustic sensors^60^ or akinetic Fabry-Perot cavities^61^, together with customized sample holders, may permit reflection mode PAM in the OC-PAM system in the future. The axial resolution of PAM (61.8 µm) is one order of magnitude larger than that of the OCM (2.68 µm). In addition, strong echoes were observed in PAM’s A lines, making thresholding a necessity to better visualize the PAM image in 3D. From Fig. 8 and the supplementary videos, we can see that OCM and the thresholded PAM can be accurately co-registered using the melanoma cells on the spheroid surfaces. However, 100% 3D co-registration between the two modalities was not achievable in this work, which could be attributed to the following reasons: first, melanin expression was not guaranteed in all melanocytes, and even if there was melanin expression, the expressed melanin could not fill the whole cell. This effect can be seen in Fig. 6 and could explain why there was no photoacoustic signal in the bottom regions of the spheroids. Second, shadow artifacts played a role in PAM imaging. Once the photon energy got fully absorbed by melanin distributed closer to the excitation beam, we could not resolve the deep-seated melanin. We believe that the hollow regions shown in Fig. 8e can be explained by this type of shadow artifact. Third, the thresholding procedure in PAM was based on amplitude values, which means that weak signals generated by melanin could have been canceled by thresholding. Higher frequency transducers and multi-angle photoacoustic detection methods may mitigate the issues mentioned above. When profusion of information outweighs high-throughput screening, a closed chamber for the imaging of individual organoids using not only OC-PAM, but also multiphoton microscopy may be envisioned^62^. On the other hand, when longitudinal high-throughput imaging is needed, for example in the application of drug screening, galvanometer mirror scanning of individual wells can be combined with long travel range motorized stages to enable batch OC-PAM imaging of 96-well plates.

More specifically on image acquisition time, the current OC-PAM configuration uses galvanometer scanning mirrors for laser beam scanning, whereas the depth information is inherently encoded in the A line. For standard confocal microscopy and multiphoton microscopy, they rely on similar laser scanning methods to scan the field of view, whereas the depth scanning is achieved by Z-stacking^63,64^. All these three methods are limited in speed by similar laser scanning mechanisms while OC-PAM has the advantage of not needing Z-stacking. Light-sheet microscopy, on the other hand, can acquire volumetric images much faster because no point-scanning is needed^65^. Although the major advantage of OC-PAM is in its label-free feature instead of in its imaging speed compared with the fluorescence-based microscopy modalities, it is worth noting that continuous efforts have been made to further increase the imaging speed of OCM^27^ and PAM^66^.

The OC-PAM system and AI-based analysis presented in this study are designed to be scalable and adaptable to various cancer research settings, from basic biology studies to drug screening applications. Future work will focus on streamlining the imaging and analysis workflow to facilitate the widespread adoption of these tools in the cancer research community. Additionally, to expand the endogenous contrast mechanism of OCM using the logarithmic intensity variance, D-OCT could be used to assess further drug-response patterns. Finally, knowing that in many applications large organoids with a few millimeter diameters are used, deeper penetration depth of the OC-PAM system will be needed to expand its applicability to study such large organoids. To achieve this, the sample arm light may be reconfigured in Bessel beam to achieve an extended focus^67^. From another perspective, instead of using conventional well plates, the samples can be levitated by acoustic trapping for orientation control, so that the imaging depth can be doubled with improved spatial resolution^68^.

In summary, we have demonstrated a multimodal OC-PAM system for the imaging of cancer organoids and cancer spheroids. The two imaging modalities are both non-invasive, label-free, and provide sub-cellular resolution with deep imaging penetration depth. The high imaging speed makes the imaging system suitable for longitudinal studies and the associated image analysis algorithms can track individual organoids precisely and measure their changes quantitatively. Organoid viability can be classified automatically using an AI-based algorithm, showing the potential for assessing the treatment efficacy of anti-cancer drugs. Using melanin as a mock-up endogenous contrast for DTP cells, rare cells with just 1% presence can be detected in 3D successfully using OC-PAM, proving its feasibility in cancer research *a fortiori*. Upon further validation, OC-PAM may serve as an innovative and powerful imaging tool for a variety of cancer organoids and cancer spheroids.

## Materials and methods

### The OC-PAM system

**Fig. 9:**
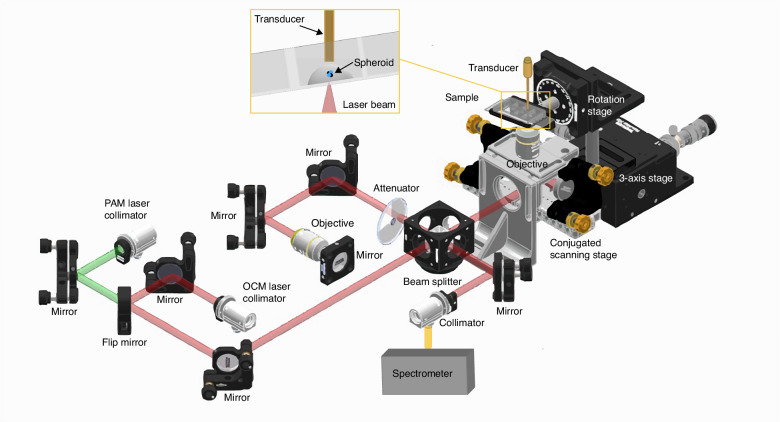
Schematic of the OC-PAM system with a spheroid sample

Figure 9 shows the schematic of the OC-PAM system developed for this project. The setup combines a spectral domain OCM sub-system with a transmission mode PAM sub-system. The imaging mode can be switched by a flip mirror. A 3SLED source (EBD290002, EXALOS) was used for the OCM mode featuring 131 nm bandwidth centered at 845 nm. The power incident upon the sample was measured to be 1.53 mW. The PAM excitation source (SPOT-10100-532, Elforlight) emitted at 532 nm at a repetition rate of 10 kHz. The pulse energy after the objective was measured to be 50 nJ for the spheroid imaging and 46 nJ for the 2D cell culture imaging. The sample arm of OCM and the excitation beam of PAM shared the same optical path and impinged upon a conjugated scanning system described in our previous work^69^, which consisted of a pair of galvanometer scanning mirrors (CTI6220H, Cambridge Technology) and two spherical mirrors. An inverted microscope design was employed for OC-PAM with the 8-well chamber slide held by a customized holder. This holder was mounted to a rotational stage (RP01/M, Thorlabs) to introduce a small angle (10 degrees) so that the incident scanning beam entered the sample not perpendicularly with respect to the bottom plate of the slide. This was necessary to suppress the back-reflection caused by the flat surface of the slide. The rotational stage was held by a translation stage (MAX313D/M, Thorlabs) for precise sample positioning and focusing. The interferogram of OCM was detected by a custom-made spectrometer and transferred to a frame grabber (PCIe-1433, National Instruments) described in a previous work^70^, whereas the photoacoustic signal was acquired using an ultrasound transducer (Optosonic Inc.) with a central frequency of 30 MHz and a −6 dB bandwidth of 16.5 MHz. This transducer was placed 2–3 mm above the sample and was co-aligned with the excitation beam during PAM imaging. The captured photoacoustic signal was amplified by a preamplifier (AU-1525, Narda-MITEQ) before being digitized by a data acquisition card (ATS9350, Alazar Technologies Inc., a time sampling rate of 250 MS/s was used). Synchronization between the lasers, scanning mirrors, and data acquisition was achieved using an FPGA (PCIe-7830, National Instruments).

The OCM sub-system achieved a lateral resolution of 3.4 µm and an axial resolution of 2.68 µm assuming 1.38 as the refractive index for soft tissue. Because of the shorter wavelength used in PAM, its lateral resolution was measured to be 1.9 µm. The acoustically determined axial resolution of PAM was measured to be 61.8 µm. For longitudinal imaging of breast cancer organoids, we used a step size of 2.52 µm to reduce the acquisition time. For 2D cell culture and spheroid imaging, we used a step size of 0.84 µm to guarantee sufficient sampling. For a typical field of view covering 0.38 mm $\times$0.34 mm for the spheroid imaging, the line-scan camera (20 kHz) limited speed of OCM yielded an image acquisition time of 9 s, and the repetition rate (10 kHz) limited speed of PAM required 18 s to finish the scan. For longitudinal imaging of breast cancer organoids, an OCM acquisition covering a 3 mm $\times$ 1.8 mm field of view took 84 s (10 kHz camera line-scan rate). In this work, lower OCM camera line-scan rate and PAM laser repetition rate were chosen for higher sensitivity.

### Image reconstruction

The OCM image was reconstructed following a standard procedure for spectral domain OCM, namely background subtraction, rescaling, and inverse Fourier transform^26^. Because the sample arm and reference arm are matched in optical pathlength by using the same components in both arms, the dispersion was largely compensated. The residual dispersion was compensated for in software. Since OCM and PAM need to be performed sequentially, fusing the volumetric images from these two modalities requires calibration. To do so, we printed a pyramid phantom (Fig. S7) with its surface coated with gold. This phantom was then imaged using OCM and PAM and the reconstructed 3D phantom images were used to extract the offset and scaling factors between OCM and PAM so that image fusion can be achieved (Fig. S8). The 3D OC-PAM image visualization was done using a workflow established in Amira (version 2023.1.1, Thermo Fisher Scientific). The centroids of melanoma cells in the spheroids were found using PAM’s peak amplitude followed by thresholding (87% of the peak amplitude). The transparency of the OCM volumes was adjusted to better demonstrate the internal melanoma cells.

### Breast cancer organoids

Murine breast cancer organoids of the KB1P model were cultivated in 10 µL droplets of basal membrane extract (BME) mixed at 1:1 ratio with advanced Dulbecco’s modified Eagle medium F12 (DMEM F12). We added 10 mM HEPES, 1x GlutaMAX, 1% penicillin-streptomycin (pen/strep), 125 µM N-acetylcysteine, B-27, 50 ng/ml epidermal growth factor (EGF), 10% of Noggin and R-spondin conditioned medium as supplements. Following trypsinization with TripleE, 1000 cells/well were seeded in 10 µL BME:media droplet in 8-well chamber slides (ibidi, 80806) which were covered with media after solidification at 37 °C for 30 min. After 3 days of culture, the organoids were separated into a control group and a treatment group with the latter one receiving 8 µM carboplatin for 5 days before the media was exchanged back to be carboplatin free. From day 3 onwards, control and treated organoids were imaged every other day for 13 and 21 days, respectively, by OCM and brightfield microscopy (IX83, Olympus). The control group was imaged until day 13 because the organoids grew beyond the maximum field of view.

### Organoid tracking

The OCM volumes of breast cancer organoids were automatically processed and segmented using a previously developed segmentation algorithm^45^. Briefly, the volumes were pre-processed using histogram normalization, stretching, and noise reduction methods. The pre-processed volumes were segmented using a deep learning segmentation network based on the K-Net, an encoder-decoder network with a shifted window (Swin) transformer backbone and a kernel generation decoder head^71^. The Swin Transformer backbone efficiently extracts multiscale features from each input, capturing long-range dependencies in 2D images. Its hierarchical structure also makes it suitable for potential future scaling to different image resolutions and complexities (e.g., fine-tuning for various types of biological organoids). The full evaluation of the method was reported previously^45^, showing an average performance of above 80% for the Dice coefficient for the segmentation of organoids past day 5.

Following segmentation, single organoids were tracked over 21 days for treated samples and 13 days for untreated samples. The tracking approach was based on a middle day reference volume selection (e.g., day 9) and then a dual branch analysis, analyzing the subsequent volume forward in time (e.g., day 11) and backward in time (e.g., day 7). The organoids were matched by using a key attribute evaluation and probability scoring based on spatial and morphological characteristics, which was previously validated^45^. The growth pattern of the different organoid wells was analyzed by differentiating between the treated samples and the untreated samples. Both an average growth and a single organoid relative growth pattern were analyzed by first computing the volume of the organoids as the sum of the segmented pixels and multiplying this by the pixel dimensions, and then by using a percentage growth analysis, to demonstrate the effectiveness of the treatment and the presence of cells with DTP-like growth patterns in the treated samples. We chose average volume as the primary growth metric as it (i) captures macroscopic changes in organoid structure that are biologically meaningful and visible in OCM data; (ii) is robust to local morphological noise compared to other shape descriptors; and (iii) allows for inter-organoid comparison and supports longitudinal tracking of growth trends.

### Viability classification

One thousand breast cancer cells were seeded into 8-well chamber wells as described in the section above. On day 3, carboplatin of increasing concentrations (0, 8 µM, 1000 µM) was added to the wells for 5 days. On day 8, the drug was removed, and the organoids were cultivated further for 13 days. From day 5 to day 15, a subset of wells from each of the three treatment groups was imaged by OCM. Following OCM scanning, organoids were labeled by acridine orange and propidium iodide (Cyto3D Live-Dead Assay Kit, The Well) using 3 µL of Cyto3D reagent per 150 µL media covering the organoid dome per well to distinguish live and dead cells by FLM (LSM980, Leica; IXplore SpinSR, Olympus). Fluorescence images were processed using local contrast-limited adaptive histogram equalization and Otsu thresholding in FIJI^72^. The viability score of the organoid was calculated as a percentage by dividing the number of pixels showing acridine orange (living cells) by the sum of all pixels showing propidium iodide (dead cells) and living cells. Organoids were given two statuses depending on a cut-off of 50% on the viability score, above which as “high viability” (*n* = 214) and below which as “low viability” (*n* = 152). Then, a radiomics-based approach was developed to differentiate organoids’ viability status in OCM volumes. Thirty-two radiomics texture parameters were computed on each single organoid on the original non-preprocessed OCM data, which were isolated thanks to the obtained segmentation mask using the same method described in the previous section. The original non-preprocessed OCM data was employed for texture feature extraction to ensure that the structural information captured by the texture descriptors remains intact and is not influenced by any processing-related artifacts. In particular, 9 first-order histogram-based features were computed. Then, second-order texture-based features were computed. These second-order features are based on the gray level co-occurrence matrix (13 features) and the gray level run length matrix (10 features). The mathematical definition of the texture features can be found in the Python Pyradiomics library^73^. The radiomics texture features computed on 37 OCM volumes acquired from 16 wells were employed to train and validate an XGBoost classifier^74^, using 10-fold cross validation. The classification results are reported using a ROC analysis and computing the AUC.

Finally, the viability analysis was implemented on the longitudinally tracked organoids described in the previous section. Again, texture features were computed on the segmented and tracked organoids and the trained classifier was employed to predict the viability status (i.e., high viability or low viability) of the organoids at the different time points and an overall viability of each tracked organoid was computed, where 0 means the organoid was always classified as having low viability, and 1 means that the organoid was always classified as having high viability. It is important to underline that the ground truth fluorescence data is not available for these volumes, as fluorescence labeling might have altered the growth kinetics and viability which is the reason for using a parallel set for classification as described above.

### 2D cell cultures

B16-F10 (ATCC) and 4T1 (ATCC) cells were cultivated in DMEM supplemented with 10% FBS and 1% pen/strep. After cultivation, each chamber of the 8-well chamber slides (80806, ibidi) was seeded either with 1000 cells of B16-F10 or 4T1.

### Co-cultured spheroids

Various ratios of B16-F10 cells and 4T1 cells were grown in DMEM supplemented with 10% FBS and 1% pen/strep. One thousand cells per spheroid were seeded into the wells of a round bottom 96-well plate and then centrifuged at 1000 rpm for 10 min. Afterwards, spheroids were allowed to grow for 3 days in an incubator at 37 °C and 5% CO_2_. Finally, spheroids were embedded in 10 µL BME: DMEM (1:1) media droplet of an 8-well chamber slide (80806, ibidi) before OC-PAM imaging and covered with DMEM with 10% FBS and 1% pen/strep.

## Supplementary information

Supplementary information for optical coherence photoacoustic microscopy for 3D cancer model imaging with AI-assisted organoid analysis

0 melanoma cell in the spheroid

1% melanoma cell in the spheroid

20% melanoma cell in the spheroid

100% melanoma cell in the spheroid

## Data Availability

All data are available upon request.

## References

[1] Gunti, S. et al. Organoid and spheroid tumor models: techniques and applications. *Cancers* **13**, 874, 10.3390/cancers13040874 (2021). 33669619 10.3390/cancers13040874 PMC7922036

[2] Zhao, Z. X. et al. Organoids. *Nat. Rev. Methods Prim.* **2**, 94, 10.1038/s43586-022-00174-y (2022). 10.1038/s43586-022-00174-y PMC10270325 37325195

[3] Pinto, B. et al. Three-dimensional spheroids as in vitro preclinical models for cancer research. *Pharmaceutics* **12**, 1186, 10.3390/pharmaceutics12121186 (2020). 33291351 10.3390/pharmaceutics12121186 PMC7762220

[4] Quereda, V. et al. A cytotoxic three-dimensional-spheroid, high-throughput assay using patient-derived glioma stem cells. *SLAS Discov.* **23**, 842–849, 10.1177/2472555218775055 (2018). 29750582 10.1177/2472555218775055 PMC6102052

[5] Lancaster, M. A. et al. Cerebral organoids model human brain development and microcephaly. *Nature* **501**, 373–379, 10.1038/nature12517 (2013). 23995685 10.1038/nature12517 PMC3817409

[6] Halfter, K. et al. Testing chemotherapy efficacy in HER2 negative breast cancer using patient-derived spheroids. *J. Transl. Med.* **14**, 112. 10.1186/s12967-016-0855-3 (2016). 27142386 10.1186/s12967-016-0855-3 PMC4855689

[7] Sachs, N. et al. A living biobank of breast cancer organoids captures disease heterogeneity. *Cell* **172**, 373–386.e10, 10.1016/j.cell.2017.11.010 (2018). 29224780 10.1016/j.cell.2017.11.010

[8] Della Corte, C. M. et al. Antitumor activity of dual blockade of PD-L1 and MEK in NSCLC patients derived three-dimensional spheroid cultures. *J. Exp. Clin. Cancer Res.* **38**, 253, 10.1186/s13046-019-1257-1 (2019). 31196138 10.1186/s13046-019-1257-1 PMC6567578

[9] Barkauskas, C. E. et al. Lung organoids: current uses and future promise. *Development* **144**, 986–997, 10.1242/dev.140103 (2017). 28292845 10.1242/dev.140103 PMC5358104

[10] Jeppesen, M. et al. Short-term spheroid culture of primary colorectal cancer cells as an in vitro model for personalizing cancer medicine. *PLOS ONE* **12**, e0183074, 10.1371/journal.pone.0183074 (2017). 28877221 10.1371/journal.pone.0183074 PMC5587104

[11] van de Wetering, M. et al. Prospective derivation of a living organoid biobank of colorectal cancer patients. *Cell* **161**, 933–945, 10.1016/j.cell.2015.03.053 (2015). 25957691 10.1016/j.cell.2015.03.053 PMC6428276

[12] Tomás-Bort, E. et al. 3D approaches to model the tumor microenvironment of pancreatic cancer. *Theranostics* **10**, 5074–5089, 10.7150/thno.42441 (2020). 32308769 10.7150/thno.42441 PMC7163433

[13] Li, X. N. et al. Oncogenic transformation of diverse gastrointestinal tissues in primary organoid culture. *Nat. Med.* **20**, 769–777, 10.1038/nm.3585 (2014). 24859528 10.1038/nm.3585 PMC4087144

[14] Hradecká, L. et al. Segmentation and tracking of mammary epithelial organoids in brightfield microscopy. *IEEE Trans. Med. Imaging* **42**, 281–290, 10.1109/TMI.2022.3210714 (2023). 36170389 10.1109/TMI.2022.3210714

[15] Kok, R. N. U. et al. Label-free cell imaging and tracking in 3D organoids. *Cell Rep. Phys. Sci.* **6**, 102522. 10.1016/j.xcrp.2025.102522 (2025).

[16] Steinbauer, S. et al. Enhanced bioluminescence imaging of tumor cells surviving chemotherapy in a murine model of triple-negative breast cancer. *npj Breast Cancer* **11**, 80. 10.1038/s41523-025-00795-y (2025). 40738896 10.1038/s41523-025-00795-y PMC12311029

[17] Drakhlis, L. et al. Human heart-forming organoids recapitulate early heart and foregut development. *Nat. Biotechnol.* **39**, 737–746, 10.1038/s41587-021-00815-9 (2021). 33558697 10.1038/s41587-021-00815-9 PMC8192303

[18] Deben, C. et al. Development and validation of the Normalized Organoid Growth Rate (NOGR) metric in brightfield imaging-based assays. *Commun. Biol.* **7**, 1612, 10.1038/s42003-024-07329-5 (2024). 39627437 10.1038/s42003-024-07329-5 PMC11615385

[19] Maharjan, S. et al. Advanced 3D imaging and organoid bioprinting for biomedical research and therapeutic applications. *Adv. Drug Deliv. Rev.* **208**, 115237. 10.1016/j.addr.2024.115237 (2024). 38447931 10.1016/j.addr.2024.115237 PMC11031334

[20] Dietrich, B. et al. NOTCH3 signalling controls human trophoblast stem cell expansion and differentiation. *Development* **150**, dev202152. 10.1242/dev.202152 (2023). 37905445 10.1242/dev.202152

[21] Noh, S. et al. Structural analysis of cerebral organoids using confocal microscopy and transmission/scanning electron microscopy. *Microsc. Microanalysis* **31**, ozae119. 10.1093/mam/ozae119 (2025). 10.1093/mam/ozae119 39999189

[22] Yildirim, M. et al. Label-free three-photon imaging of intact human cerebral organoids for tracking early events in brain development and deficits in Rett syndrome. *eLife* **11**, e78079. 10.7554/eLife.78079 (2022). 35904330 10.7554/eLife.78079 PMC9337854

[23] Jain, A. et al. Morphodynamics of human early brain organoid development. *Nature* **644**, 1010–1019, 10.1038/s41586-025-09151-3 (2025). 40533563 10.1038/s41586-025-09151-3 PMC12390842

[24] Dekkers, J. F. et al. High-resolution 3D imaging of fixed and cleared organoids. *Nat. Protoc.* **14**, 1756–1771, 10.1038/s41596-019-0160-8 (2019). 31053799 10.1038/s41596-019-0160-8

[25] Hofbauer, P. et al. Cardioids reveal self-organizing principles of human cardiogenesis. *Cell* **184**, 3299–3317.e22, 10.1016/j.cell.2021.04.034 (2021). 34019794 10.1016/j.cell.2021.04.034

[26] Drexler, W. et al. Optical coherence tomography today: speed, contrast, and multimodality. *J. Biomed. Opt.* **19**, 071412, 10.1117/1.JBO.19.7.071412 (2014). 25079820 10.1117/1.JBO.19.7.071412

[27] Leitgeb, R. et al. Enhanced medical diagnosis for dOCTors: a perspective of optical coherence tomography. *J. Biomed. Opt.* **26**, 100601, 10.1117/1.JBO.26.10.100601 (2021). 34672145 10.1117/1.JBO.26.10.100601 PMC8528212

[28] Zhang, L. et al. Oblique scanning laser microscopy for simultaneously volumetric structural and molecular imaging using only one raster scan. *Sci. Rep.* **7**, 8591. 10.1038/s41598-017-08822-0 (2017). 28819250 10.1038/s41598-017-08822-0 PMC5561209

[29] Huang, Y. Y. et al. Optical coherence tomography detects necrotic regions and volumetrically quantifies multicellular tumor spheroids. *Cancer Res.* **77**, 6011–6020, 10.1158/0008-5472.Can-17-0821 (2017). 28904062 10.1158/0008-5472.CAN-17-0821 PMC5866924

[30] Gil, D. A., Deming, D. A. & Skala, M. C. Volumetric growth tracking of patient-derived cancer organoids using optical coherence tomography. *Biomed. Opt. Express* **12**, 3789–3805, 10.1364/BOE.428197 (2021). 34457380 10.1364/BOE.428197 PMC8367263

[31] Deloria, A. J. et al. Ultra-high-resolution 3D optical coherence tomography reveals inner structures of human placenta-derived trophoblast organoids. *IEEE Trans. Biomed. Eng.* **68**, 2368–2376, 10.1109/TBME.2020.3038466 (2021). 33201804 10.1109/TBME.2020.3038466

[32] Ming, Y. X. et al. Longitudinal morphological and functional characterization of human heart organoids using optical coherence tomography. *Biosens. Bioelectron.* **207**, 114136. 10.1016/j.bios.2022.114136 (2022). 35325716 10.1016/j.bios.2022.114136 PMC9713770

[33] Hao, S. Y. et al. Dual-modality imaging system for monitoring human heart organoids beating in vitro. *Opt. Lett.* **48**, 3929–3932, 10.1364/OL.493824 (2023). 37527085 10.1364/OL.493824 PMC10707703

[34] Abd El-Sadek, I. et al. Label-free visualization and quantification of the drug-type-dependent response of tumor spheroids by dynamic optical coherence tomography. *Sci. Rep.* **14**, 3366. 10.1038/s41598-024-53171-4 (2024). 38336794 10.1038/s41598-024-53171-4 PMC10858208

[35] Abd El-Sadek, I. et al. Label-free drug response evaluation of human derived tumor spheroids using three-dimensional dynamic optical coherence tomography. *Sci. Rep.* **13**, 15377. 10.1038/s41598-023-41846-3 (2023). 37717067 10.1038/s41598-023-41846-3 PMC10505213

[36] Hosseinaee, Z., Tummon Simmons, J. A. & Reza, P. H. Dual-modal photoacoustic imaging and optical coherence tomography [Review]. *Front. Phys.* **8**, 616618. 10.3389/fphy.2020.616618 (2021).

[37] Wang, L. V. & Yao, J. J. A practical guide to photoacoustic tomography in the life sciences. *Nat. Methods* **13**, 627–638, 10.1038/nmeth.3925 (2016). 27467726 10.1038/nmeth.3925 PMC4980387

[38] Yao, J. J., Song, L. & Wang, L. V. Photoacoustic microscopy: superdepth, superresolution, and superb contrast. *IEEE Pulse* **6**, 34–37, 10.1109/MPUL.2015.2409100 (2015). 25974913 10.1109/MPUL.2015.2409100 PMC6853069

[39] Ntziachristos, V. Going deeper than microscopy: the optical imaging frontier in biology. *Nat. Methods* **7**, 603–614, 10.1038/nmeth.1483 (2010). 20676081 10.1038/nmeth.1483

[40] Haindl, R. et al. Functional optical coherence tomography and photoacoustic microscopy imaging for zebrafish larvae. *Biomed. Opt. Express* **11**, 2137–2151, 10.1364/BOE.390410 (2020). 32341872 10.1364/BOE.390410 PMC7173920

[41] Haindl, R. et al. Dual modality reflection mode optical coherence and photoacoustic microscopy using an akinetic sensor. *Opt. Lett.* **42**, 4319–4322, 10.1364/OL.42.004319 (2017). 29088153 10.1364/OL.42.004319

[42] Zhu, X. Y. et al. Resolution-matched reflection mode photoacoustic microscopy and optical coherence tomography dual modality system. *Photoacoustics* **19**, 100188. 10.1016/j.pacs.2020.100188 (2020). 32577377 10.1016/j.pacs.2020.100188 PMC7300161

[43] Englert, L. et al. Fast 3D optoacoustic mesoscopy of neuromelanin through entire human midbrain organoids at single-cell resolution. *Laser Photonics Rev.* **17**, 2300443. 10.1002/lpor.202300443 (2023).

[44] Darrigues, E. et al. Tracking Gold Nanorods’ interaction with large 3D pancreatic-stromal tumor spheroids by multimodal imaging: fluorescence, photoacoustic, and photothermal microscopies. *Sci. Rep.* **10**, 3362. 10.1038/s41598-020-59226-6 (2020). 32099027 10.1038/s41598-020-59226-6 PMC7042370

[45] Branciforti, F. et al. Segmentation and multi-timepoint tracking of 3D cancer organoids from optical coherence tomography images using deep neural networks. *Diagnostics* **14**, 1217, 10.3390/diagnostics14121217 (2024). 38928633 10.3390/diagnostics14121217 PMC11203156

[46] Faust, O. et al. Comparative assessment of texture features for the identification of cancer in ultrasound images: a review. *Biocybern. Biomed. Eng.* **38**, 275–296, 10.1016/j.bbe.2018.01.001 (2018).

[47] Chitalia, R. D. & Kontos, D. Role of texture analysis in breast MRI as a cancer biomarker: a review. *J. Magn. Reson. Imaging* **49**, 927–938, 10.1002/jmri.26556 (2019). 30390383 10.1002/jmri.26556 PMC7077754

[48] Wang, B. J. et al. Deep learning based characterization of human organoids using optical coherence tomography. *Biomed. Opt. Express* **15**, 3112–3127, 10.1364/BOE.515781 (2024). 38855657 10.1364/BOE.515781 PMC11161340

[49] Xu, M. Z. et al. Artificial intelligence-aided optical imaging for cancer theranostics. *Semin. Cancer Biol.* **94**, 62–80, 10.1016/j.semcancer.2023.06.003 (2023). 37302519 10.1016/j.semcancer.2023.06.003

[50] Wu, J. C. et al. Learned end-to-end high-resolution lensless fiber imaging towards real-time cancer diagnosis. *Sci. Rep.* **12**, 18846. 10.1038/s41598-022-23490-5 (2022). 36344626 10.1038/s41598-022-23490-5 PMC9640670

[51] Han, G. R. et al. Machine learning in point-of-care testing: innovations, challenges, and opportunities. *Nat. Commun.* **16**, 3165. 10.1038/s41467-025-58527-6 (2025). 40175414 10.1038/s41467-025-58527-6 PMC11965387

[52] Wang, S. et al. Towards next-generation diagnostic pathology: AI-empowered label-free multiphoton microscopy. *Light Sci. Appl.* **13**, 254, 10.1038/s41377-024-01597-w (2024). 39277586 10.1038/s41377-024-01597-w PMC11401902

[53] Du, Y. et al. Hybrid multimode - multicore fibre based holographic endoscope for deep-tissue neurophotonics. *Light Adv. Manuf.* **3**, 408–416, 10.37188/lam.2022.029 (2022).

[54] Duarte, A. A. et al. BRCA-deficient mouse mammary tumor organoids to study cancer-drug resistance. *Nat. Methods* **15**, 134–140, 10.1038/nmeth.4535 (2018). 29256493 10.1038/nmeth.4535

[55] Szebényi, K. et al. Effective targeting of breast cancer by the inhibition of P-glycoprotein mediated removal of toxic lipid peroxidation byproducts from drug tolerant persister cells. *Drug Resist. Updates* **71**, 101007. 10.1016/j.drup.2023.101007 (2023). 10.1016/j.drup.2023.101007 37741091

[56] Ramirez, M. et al. Diverse drug-resistance mechanisms can emerge from drug-tolerant cancer persister cells. *Nat. Commun.* **7**, 10690. 10.1038/ncomms10690 (2016). 26891683 10.1038/ncomms10690 PMC4762880

[57] Liu, C. B. et al. Advances in imaging techniques and genetically encoded probes for photoacoustic imaging. *Theranostics* **6**, 2414–2430, 10.7150/thno.15878 (2016). 27877244 10.7150/thno.15878 PMC5118604

[58] Weber, J., Beard, P. C. & Bohndiek, S. E. Contrast agents for molecular photoacoustic imaging. *Nat. Methods* **13**, 639–650, 10.1038/nmeth.3929 (2016). 27467727 10.1038/nmeth.3929

[59] Wang, L. V. Multiscale photoacoustic microscopy and computed tomography. *Nat. Photonics* **3**, 503–509, 10.1038/nphoton.2009.157 (2009). 20161535 10.1038/nphoton.2009.157 PMC2802217

[60] Ansari, R. et al. All-optical forward-viewing photoacoustic probe for high-resolution 3D endoscopy. *Light Sci. Appl.* **7**, 75, 10.1038/s41377-018-0070-5 (2018). 30323927 10.1038/s41377-018-0070-5 PMC6177463

[61] Preisser, S. et al. All-optical highly sensitive akinetic sensor for ultrasound detection and photoacoustic imaging. *Biomed. Opt. Express* **7**, 4171–4186, 10.1364/BOE.7.004171 (2016). 27867723 10.1364/BOE.7.004171 PMC5102516

[62] Liu, M. Y. et al. REAP: revealing drug tolerant persister cells in cancer using contrast enhanced optical coherence and photoacoustic tomography. *J. Phys. Photonics* **3**, 021001, 10.1088/2515-7647/abf02f (2021).

[63] Hoover, E. E. & Squier, J. A. Advances in multiphoton microscopy technology. *Nat. Photonics* **7**, 93–101, 10.1038/nphoton.2012.361 (2013). 24307915 10.1038/nphoton.2012.361 PMC3846297

[64] Elliott, A. D. Confocal microscopy: principles and modern practices. *Curr. Protoc. Cytom.* **92**, e68. 10.1002/cpcy.68 (2020). 31876974 10.1002/cpcy.68 PMC6961134

[65] Stelzer, E. H. K. et al. Light sheet fluorescence microscopy. *Nat. Rev. Methods Prim.* **1**, 73, 10.1038/s43586-021-00069-4 (2021).

[66] Zhu, X. Y. et al. J. High speed innovations in photoacoustic microscopy. *npj Imaging* **2**, 46, 10.1038/s44303-024-00052-0 (2024). 39525278 10.1038/s44303-024-00052-0 PMC11541221

[67] Glandorf, L. et al. Bessel beam optical coherence microscopy enables multiscale assessment of cerebrovascular network morphology and function. *Light Sci. Appl.* **13**, 307, 10.1038/s41377-024-01649-1 (2024). 39523430 10.1038/s41377-024-01649-1 PMC11551179

[68] Kvåle Løvmo, M. et al. Ultrasound-induced reorientation for multi-angle optical coherence tomography. *Nat. Commun.* **15**, 2391. 10.1038/s41467-024-46506-2 (2024). 38493195 10.1038/s41467-024-46506-2 PMC10944478

[69] Deng, S. Y. et al. An optical coherence photoacoustic microscopy system using a fiber optic sensor. *APL Photonics* **6**, 096103. 10.1063/5.0059351 (2021).

[70] Haindl, R. et al. Ultra-high-resolution SD-OCM imaging with a compact polarization-aligned 840 nm broadband combined-SLED source. *Biomed. Opt. Express* **11**, 3395–3406, 10.1364/BOE.394229 (2020). 32637262 10.1364/BOE.394229 PMC7316001

[71] Zhang, W. W. et al. K-net: towards unified image segmentation. 35th International conference on neural information processing systems. 10326–10338, https://dl.acm.org/doi/10.5555/3540261.3541051 (2021).

[72] Schindelin, J. et al. Fiji: an open-source platform for biological-image analysis. *Nat. Methods* **9**, 676–682, 10.1038/nmeth.2019 (2012). 22743772 10.1038/nmeth.2019 PMC3855844

[73] van Griethuysen, J. J. M. et al. Computational radiomics system to decode the radiographic phenotype. *Cancer Res.* **77**, e104–e107, 10.1158/0008-5472.Can-17-0339 (2017). 29092951 10.1158/0008-5472.CAN-17-0339 PMC5672828

[74] Chen, T. Q. & Guestrin, C. XGBoost: a scalable tree boosting system. 22nd ACM SIGKDD International Conference on knowledge discovery and data mining. 785–794, https://dl.acm.org/doi/10.1145/2939672.2939785 (2016).

