# Supplementary material for: Optical coherence photoacoustic microscopy for 3D cancer model imaging with AI-assisted organoid analysis
Source: Light Sci Appl. 2026 Feb 5;15:106. doi: 10.1038/s41377-025-02177-2 (PMC12876881; doi:10.1038/s41377-025-02177-2)
Supplement: Supplementary file 1 — Supplementary information for optical coherence photoacoustic microscopy for 3D cancer model imaging with AI-assisted organoid analysis [file 41377_2025_2177_MOESM1_ESM.pdf]

Supplementary Information for

Optical Coherence Photoacoustic Microscopy for 3D Cancer Model Imaging with AI-Assisted Organoid Analysis

Authors and affiliations:

Abigail J. Deloria[1], Agnes Csiszar[2], Shiyu Deng[1], Mohammad Ali Sabbaghi[2], Francesco Branciforti[3], Lukasz Bugyi[1], Giulia Rotunno[3], Richard Haindl[1], Rainer Leitgeb[1], Massimo Salvi[3], Manojit Pramanik[4], Yi Yuan[5], Leopold Schmetterer[1,6,7,8,9,10,11,12], Gergely Szakacs[2], Wolfgang Drexler[1], Kristen M. Meiburger[3], and Mengyang Liu[1,7,*]

[1]Center for Medical Physics and Biomedical Engineering, Medical University of Vienna, Vienna, Austria
[2]Center for Cancer Research, Medical University of Vienna, Austria
[3]PolitoBIOMed Lab, Biolab, Department of Electronics and Telecommunications, Politecnico di Torino, Torino, Italy
[4]Department of Electrical and Computer Engineering, Iowa State University, Ames, IA, USA
[5]School of Electrical Engineering, Yanshan University, Qinhuangdao, Hebei, China
[6]Department of Clinical Pharmacology, Medical University of Vienna, Vienna, Austria
[7]Singapore Eye Research Institute, Singapore
[8]SERI-NTU Advanced Ocular Engineering (STANCE) Laboratory, Singapore
[9]Ophthalmology and Visual Sciences Academic Clinical Program, Duke-NUS Medical School, Singapore
[10]Institute of Molecular and Clinical Ophthalmology Basel, Basel, Switzerland
[11]School of Chemistry, Chemical Engineering and Biotechnology, Nanyang Technological University, Singapore
[12]Fondation Ophthalmologique Adolphe De Rothschild, Paris, France

[*]**Corresponding author:**

Mengyang Liu

mengyang.liu@meduniwien.ac.at

1. Cell seeding density

Prior to performing the longitudinal study, different seeding densities - including 2500 cells and 7500 cells - were tested. Thereby, organoids were enzymatically dissociated into single cells using TriplE (Gibco) and seeded at the indicated cell density in 10 μL BME:ENR per well. After gelation, culture medium was overlaid. Optical coherence microscopy (OCM) imaging was performed on day 7. However, in both cases, shadow artifacts caused by overlapping organoids were observed, rendering the images as inappropriate for subsequent segmentation and texture analysis. Therefore, seeding density of 1000 cells was selected as the optimal compromise between cell numbers and minimal shadow artefacts.

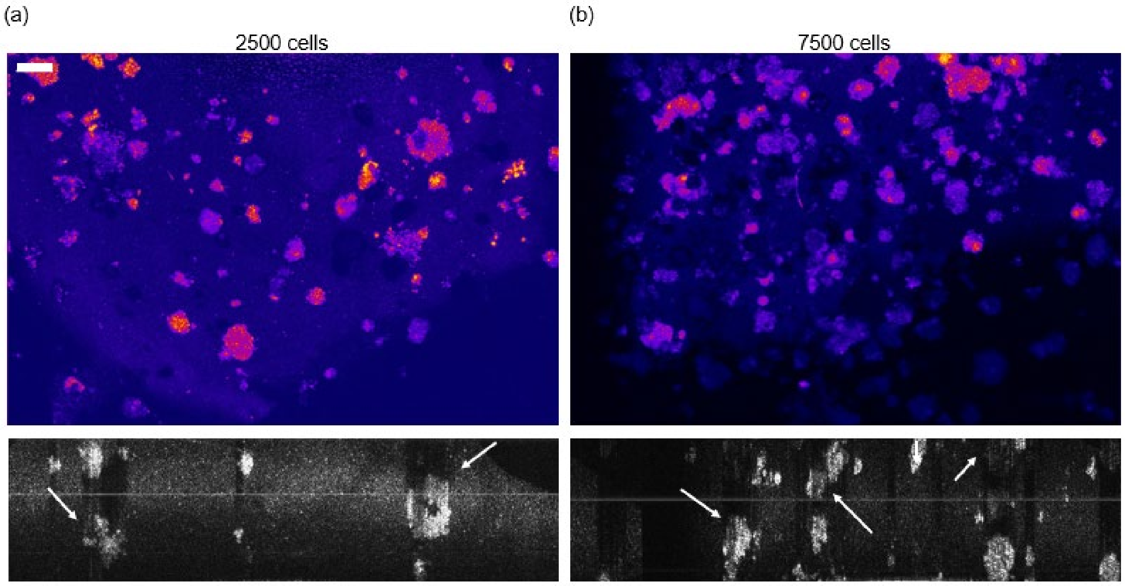

Fig. S1. Seeding density imaged with OCM using a step size of 2.52 μm covering a field of view of 3 mm × 1.8 mm. *En face* (top) and cross section (bottom) images of (a) 2500 cells (b) 7500 cells on day 7. White arrows indicate shadow artefacts. Scalebar: 200 μm.

2. Algorithm capabilities of characterizing organoid growth phenomena

In the context of long-term organoid culture, volumetric growth can be accompanied by additional biological processes such as the merging of adjacent organoids and the development of internal structures, including necrotic cores. These phenomena are important to monitor, as they can significantly influence organoid morphology, function, and interpretation of experimental outcomes. In Fig. S2 we demonstrate how the proposed algorithm can remain sensitive to structural rearrangements and internal heterogeneity.

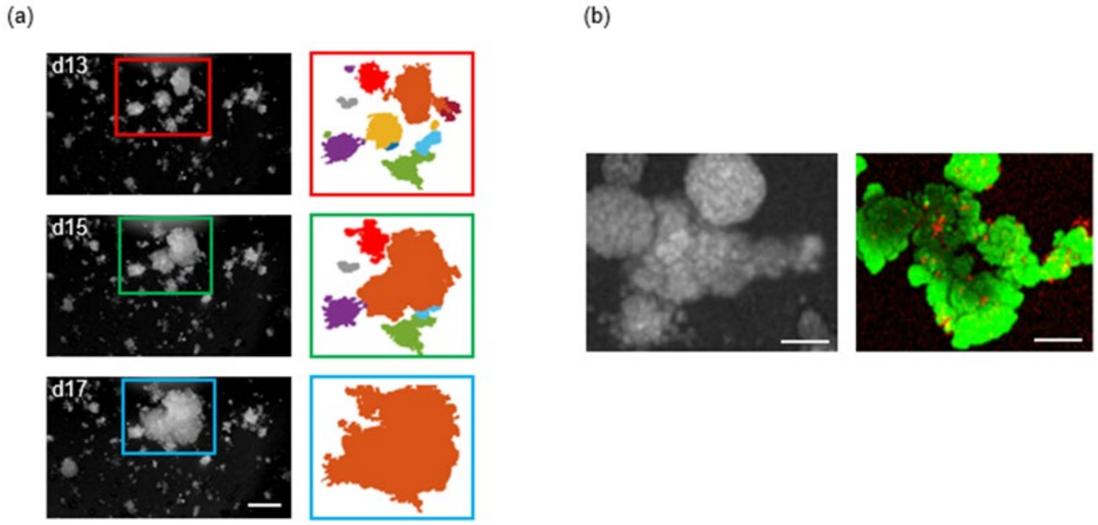

Fig. S2. Merging organoids (a) Left: OCM images of organoid culture, scalebar: 400 μm. Right: Algorithm visualizes merging of organoids. Color coding was applied to indicate different organoid IDs. When multiple organoids merge, the resulting structure adopts the ID of the largest original organoid. (b) OCM and fluorescent image of merged organoids, green indicating living cells, red necrotic cells. Scalebar: 100 μm.

Figure S2a portrays the same organoid well as presented in Fig. 2 at three specific time points, highlighting how the algorithm allows to evaluate the merging of organoids during growth. Indeed, on day 13 the organoids are all separated with different labels (i.e., colors). On day 15, it can be appreciated how the organoids presented in orange and yellow merge and take on the same label as the largest organoid. On day 17, the organoids further merge into one overarching organoid, displayed in orange. Figure S2b shows the same merged organoids imaged with OCM (left) and with FLM (right). While the cell culture dish was positioned perpendicularly during FLM imaging, a 10° tilt was introduced during OCM imaging to minimize reflection artifacts, resulting in a slight deviation in the viewing angle. Organoid culture was grown for 7 days and subsequently labeled with the dyes acridine orange (green channel) and propidium iodide (red channel) to distinguish live and dead cells, respectively. With increasing size of the 3D structure, necrotic cores (red) form.

3. Characterization of breast cancer organoids for morphology and biomarker expression.

To characterize KB1P breast cancer organoids, different light microscopy imaging technologies were used. For label-free, non-invasive and non-destructive imaging of live organoids in a 3D matrix, an Olympus CKX53 Upright Widefield Brightfield Microscope with a 10X Achromat Phasecontrast NA 0.25, 8.8 mm WD objective, a CAM-UC90 Color CCD, 9,1Mpx, USB3 detection system and cellSens 3 software was used (Fig. S3, left panels). For histopathological hematoxylin-eosin (HE) staining, organoids were first removed from the 3D cultivation matrix by several washing steps, fixed in 4% paraformaldehyde for 2 hours, embedded in low-melting agarose, dehydrated overnight in 80% ethanol followed by being embedded into paraffin blocks, getting cut into 4 μm thin sections by microtome and HE stained following a standard protocol. The slides were digitized with a Pannoramic SCAN II slide scanner (3DHistech) in extended focus scanning mode using a 20X plan-apochromat objective (0.8 NA) and a 5Mpxl sCMOS camera. The images were captured by the CaseViewer (3DHistech) software (Fig. S3, middle panels). For immunofluorescence microscopy, organoids were removed from the matrix, fixed,

immunostained for epithelial growth factor receptor (EGFR) and E-cadherin expression with DAPI counterstaining for nuclei detection, followed by clearance and embedding according to the standard protocol[1]. Images were taken using an Olympus IXplore SpinSR Spinning Disk Inverse Confocal Microscope with a 30X UPLSAPO30XS Plan Super Apochromat NA 1.05 DIC SILICONE OIL objective, Hamamatsu ORCA-FLASH sCMOS detection system, and Olympus cellSENS Dimension software (Fig. S3, right panels).

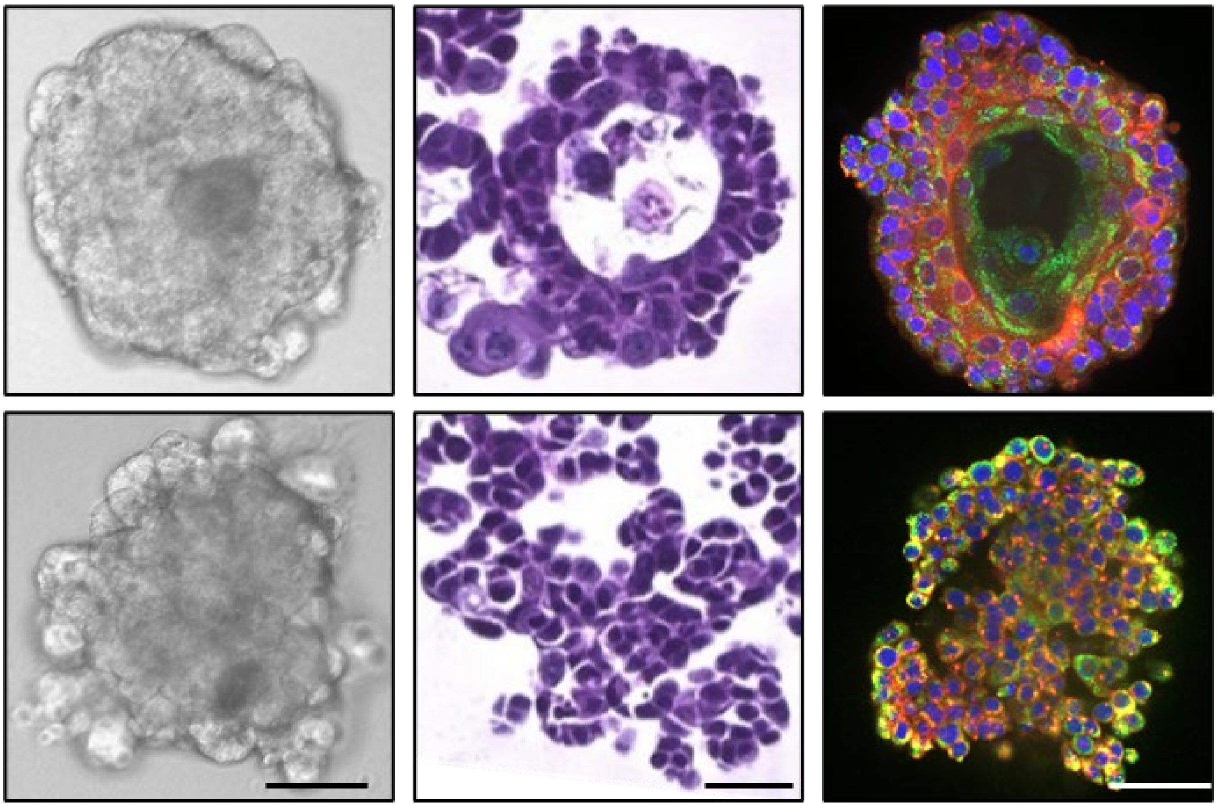

Fig. S3. Characterization of breast cancer organoids by different light microscopy imaging technologies. Left: Bright field microscopy; middle: histological analysis of HE stained thin sections of formalin-fixed paraffin embedded (FFPE) organoids; right: merged confocal microscopy images of immunofluorescent staining for epithelial growth factor receptor (EGFR, green) and E-cadherin (red) expression, nuclei counterstained by DAPI (blue). Morphological heterogeneity spans from structures with central lumen (top panel) to dense structures (lower panel). Scalebar: 50 μm.

4. Evaluation of classifier performance change with different training set sizes

More data typically implies performance improvement when considering machine learning applications, but it is not necessarily linear with sample size, especially in high-dimensional feature spaces. Indeed, beyond a certain point, more data also demands careful balancing to avoid overfitting or introducing new biases. To evaluate the classifier performance changes with different training set sizes, we employed the same ROC curve analysis using only a subset of the entire dataset, ranging from 10% to 100%, with a step size of 10%. The results are shown in Fig. S4.

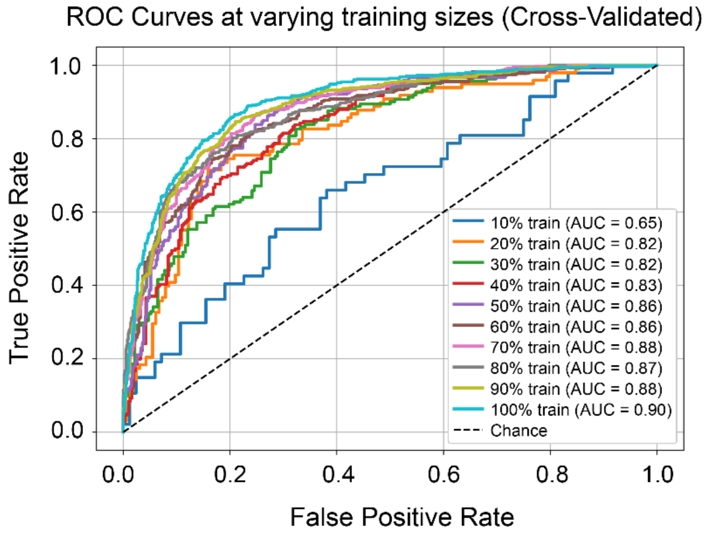

Fig. S4. ROC curve analysis using different training set sizes.

As can be seen from Fig. S4, when using different percentages of the training set for training the classifier, there is a definite decrease when a very small dataset (10%) is employed. Even at 20%, the AUC increases up to 0.82 and then slowly increases up until 0.90 when employing the entire dataset. This shows that classification performance begins to level off; although expanding the dataset remains important and can further improve the results, the task itself is inherently challenging, and a classification performance plateau can be expected.

5. Viability comparison between treated and untreated organoid wells

An important aspect of the present study is the assessment of organoid viability, which reflects the overall health and functional integrity of the culture. While the longitudinal analysis and classification results on the treated and untreated wells lack the reference data to completely validate the results, the overall findings are very encouraging.

Figure S5 displays qualitative and quantitative results of the obtained organoid viability classification results on untreated and treated wells. In panel (a) of Figure S5 a heatmap presenting the overall viability status of the untreated and treated wells are portrayed. To compute the viability status, the classification output (0 for low viability status and 1 for high viability status) of each tracked organoid was averaged at each time point. The obtained values are then visualized using the portrayed colormap. What can be observed here is that the treated well typically portrays lower average viability when compared to the untreated well, especially at days 11 and day 13. Then, the treated well shows a minimum value on day 15, after which the average viability status of the organoids starts to increase. Interestingly, this time point (i.e., between day 15 and day 17) coincides with when the organoids begin portraying an exponential increase in average volume as well (Figure 3 of the main manuscript). Panel (b) of Figure S5 instead portrays an example volume rendering of the considered untreated and treated wells with 4 single organoids highlighted. The volume growth of these organoids is then portrayed on the right and the average organoid viability score for each organoid is reported. The average organoid viability score was computed as the average of the classifier output over the considered time points (e.g., an average viability score equal to 1.0 means that the organoid was classified as having a high viability status at all considered time points).

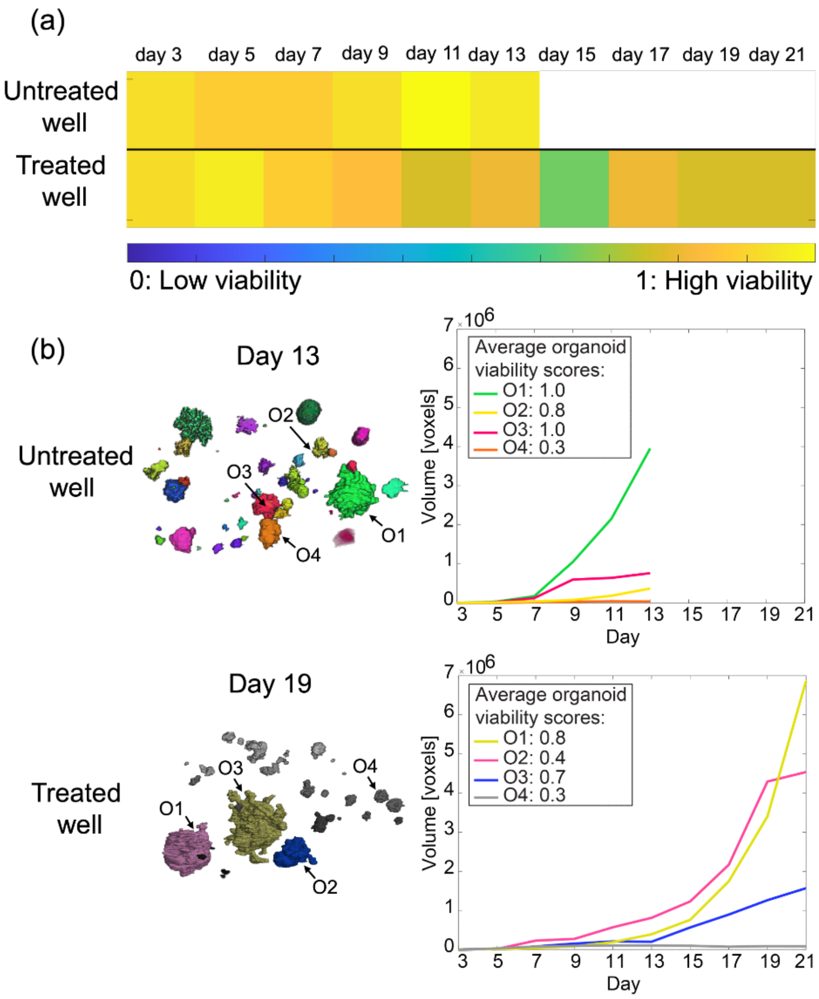

Fig. S5. Viability comparison between untreated and treated wells. (a) Heatmap representation of the average viability status of untreated and treated wells at the considered time points. (b) Select representation of four organoids (O1, O2, O3 and O4) for one untreated and one treated well portraying the single organoid average volume increase and the average organoid viability score.

6. Correlation between PAM signal and melanin

To establish a proper correlation between the PAM signal and melanin concentration, we performed PAM imaging of a tubing phantom filled with different concentrations of melanin. In this experiment, synthetic melanin (Sigma-Adrich, M8631) was diluted in distilled water. Six concentrations were used in the experiment, and their absorbance is given in Fig. S6a. The melanin solution was injected into a silicon tube (SANSYO 94-2703) with an inner diameter of 0.2 mm and an outer diameter of 0.3 mm. A syringe pump (Harvard Apparatus 11 Pico Plus Elite) was used to ensure a flow rate of 1 μLmin^{-1} to minimize sedimentation. Figure S6b shows a photo illustrating the experiment configuration with the needle transducer above the phantom. All measurements used 532 nm excitation with 46 nJ pulse energy measured at the sample surface. The excitation laser repetition rate was set at 10 kHz. Photos of various concentrations of the melanin solution in multi-well plates are given in Fig. S6c. The PAM experiment results with the unified dynamic range are given in Fig. S6d in the upper row. The lower image of Fig. S6d is a plot showing the correlation between PAM signal amplitude and melanin concentration, measured using the results shown in the upper row.

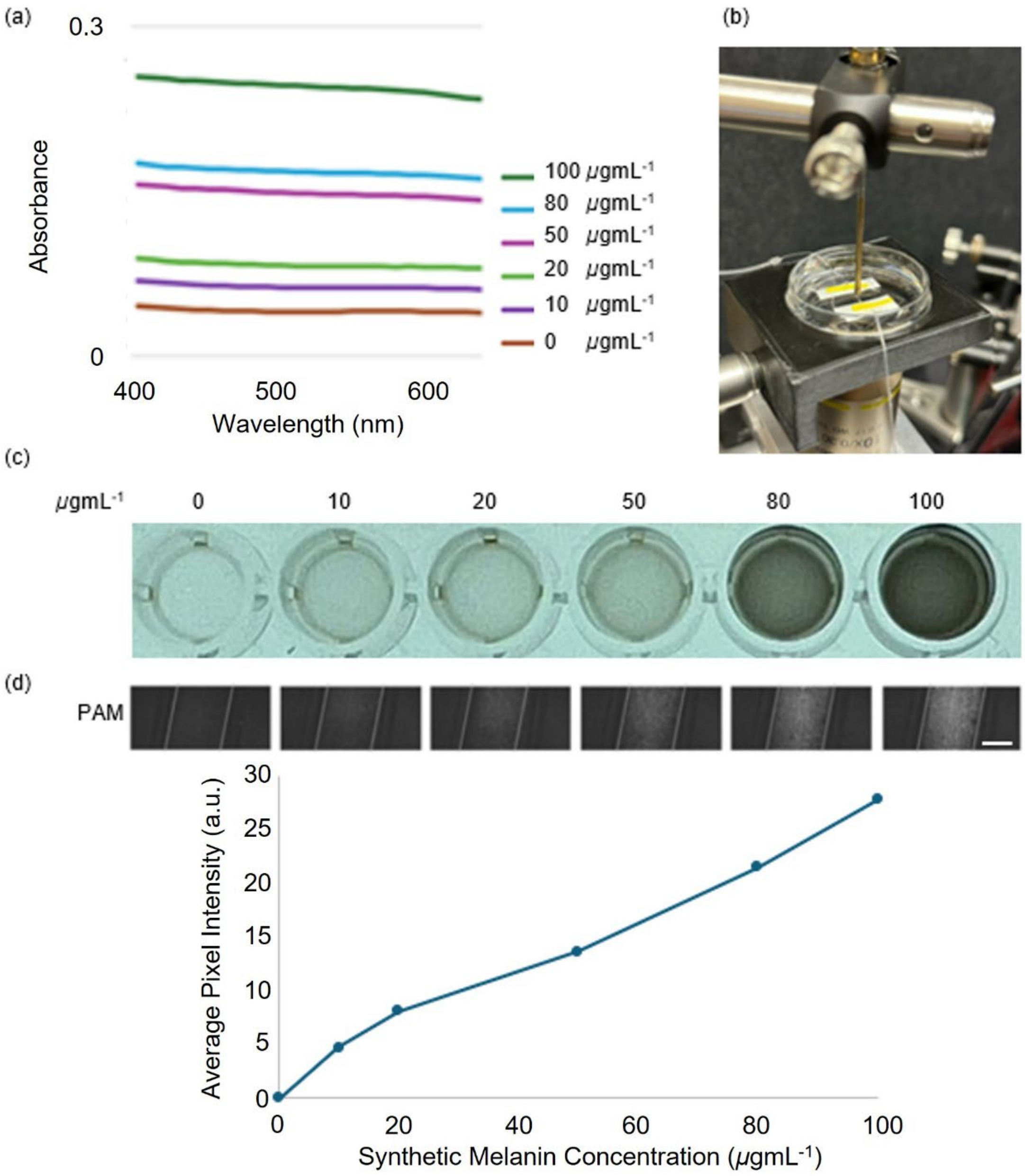

Fig. S6. (a) Absorbance of the synthetic melanin solution at various concentrations. (b) A photo showing the tubing phantom and the needle transducer during PAM measurement. (c) Photo of multi-well plates with different wells filled with melanin solution at various concentrations. (d) Upper row: PAM imaging results of the melanin solution filled tubing phantom. Scalebar 100 μm. Lower row: using the results in the upper row, this plot shows the correlation between the PAM signal amplitude (represented as the average pixel intensity) and the melanin concentration.

7. Phantom for OC-PAM image fusion

In order to verify alignment of PAM and OCM imaging volumes, a spatial synthetic phantom was needed. The shape chosen was a stepped pyramid, due to easily recognizable surfaces normal to all dimensions. The phantom was designed in CAD software (Autodesk Fusion, Autodesk Inc. USA) as a quarter of stepped, hollow pyramid (2.8 mm × 2.1 mm × 1.6 mm). The shape features 7 levels of equal width, depth, and height with a wall thickness of 120 μm. The vertical walls are angled at 15 degrees outwards to facilitate supportless the 3D printing process. The phantom was 3D-printed using a 2-photon process (NanoOne 1000, UpNano GmbH, Austria) using 10x objective out of general-purpose resin (UpPhoto, UpNano GmbH, Austria). To enable PAM contrast, the phantom was uniformly coated with a thin layer of

sputtered gold (Quorum Q150R ES, Quorum Technologies Ltd. United Kingdom). Since the 3D cancer model used in the work was embedded in Matrigel, we also embedded this 3D printed phantom in Matrigel and placed it into a multi-well plate for all OC-PAM imaging experiments. Figure S7 shows the design and microscopic images of the phantom.

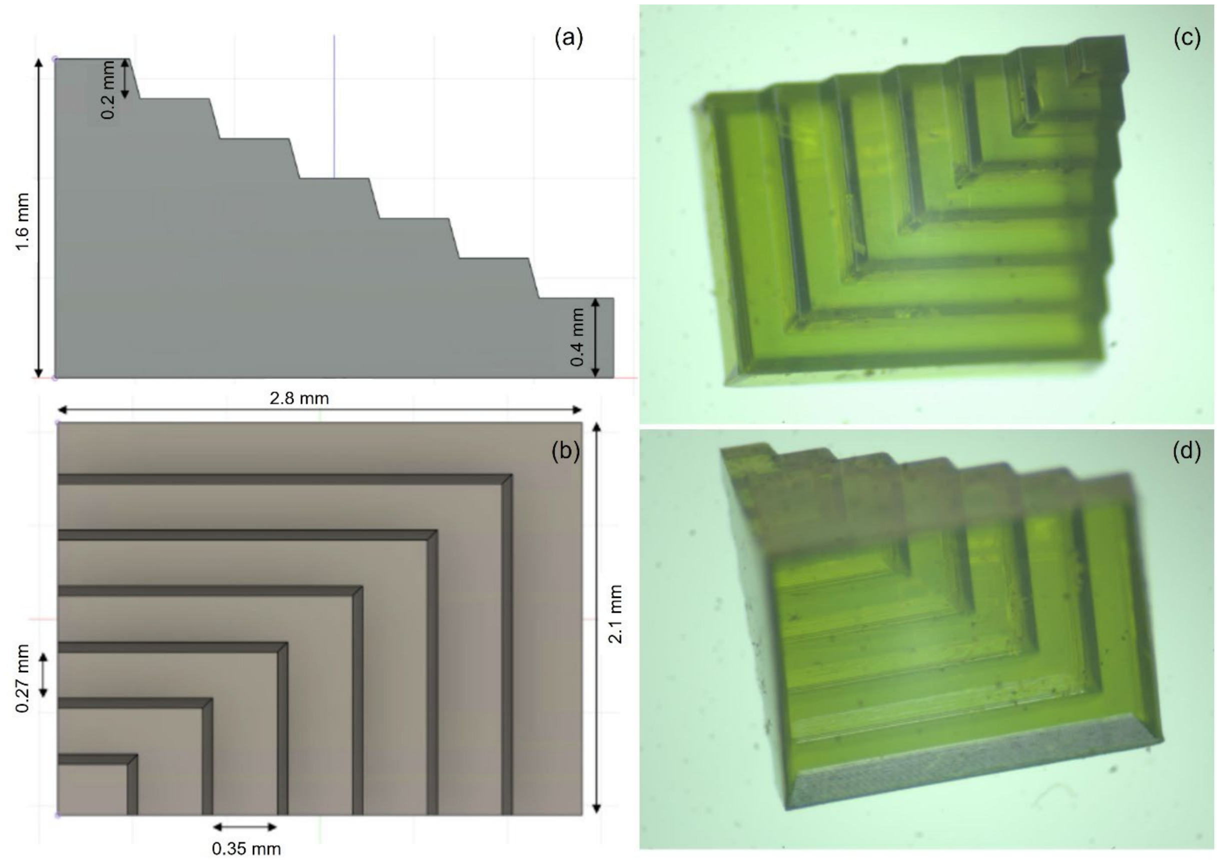

Fig. S7. (a) Side view of the phantom schematic. (b) Top view of the phantom schematic. (c) and (d), microscopic images of the phantom.

Setting the phantom at a fixed position, OCM and PAM imaging were performed sequentially. The image reconstruction was done separately for these two imaging modalities and the initially reconstructed volumes were then loaded in Amira (Thermo Fisher Scientific, version 2025.1). Using gray to represent OCM image and gold to represent PAM image, Fig. S8a and Fig. S8b shows the 3D OCM and PAM volume, respectively. Because the pyramid's dimensions are known a priori, we can use the designed dimensions to individually calculate the scaling factors for OCM and PAM images. Once the OCM and PAM volume are matched in voxel dimensions, they are merged into one fused volume as shown in Fig. S8c and Fig. S8d. The image offset between OCM and PAM can be calculated when the two volumes are merged in Amira in 3D. Evaluating from Fig. S8d, we can see that the imaging OCM was able to show 4 layers of the pyramid, indicating an imaging depth > 600 μm, whereas APM can show 3 layers of the pyramid properly, indicating an imaging depth > 400 μm.

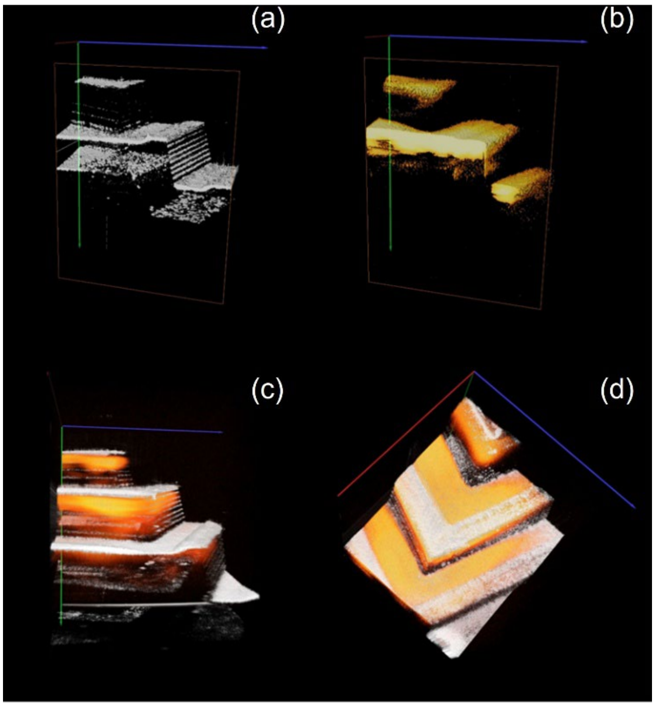

Fig. S8. (a) 3D visualization of OCM image of the phantom (clipped view). (b) 3D visualization of PAM image of the phantom. (c) and (d), two perspectives of the fused OC-PAM 3D visualization of the phantom. Gray: OCM channel; gold: PAM channel.

References

1. Dekkers, J.F., Alieva, M., Wellens, L.M. *et al.* High-resolution 3D imaging of fixed and cleared organoids. *Nat Protoc* **14**, 1756–1771 (2019). https://doi.org/10.1038/s41596-019-0160-8
